# A COBRA‐like protein ACL83 acts downstream of transcription factor ROC5 to control abaxial leaf curling in rice

**DOI:** 10.1111/tpj.71144

**Published:** 2026-09-28

**Authors:** Congping Chen, Rui Li, Jiahao Deng, Hongshan Zhang, Dan Liu, Yongkang Xiao, Junjie Liu, Hao Shu, Yue Liu, Xiaorong Yang, Jia Guo, Ying Zhong, Bin Yang, Changhui Sun, Xiaojian Deng, Pingrong Wang

**Affiliations:** ^1^ State Key Laboratory of Crop Gene Exploration and Utilization in Southwest China Sichuan Agricultural University Chengdu Sichuan China; ^2^ Rice Research Institute Sichuan Agricultural University Chengdu Sichuan China

**Keywords:** rice (*Oryza sativa*), leaf rolling, bulliform cell, COBRA‐like protein, transcription factor ROC5

## Abstract

Leaf rolling is considered an important agronomic trait in rice (*Oryza sativa*). Moderate leaf rolling contributes to erect leaves and a compact plant architecture, thereby improving photosynthetic efficiency and grain yield. However, the molecular mechanisms regulating leaf rolling remain incompletely understood. In this study, we isolated an *acl83* mutant in *indica* rice, which exhibits abaxially curled leaves due to increased area of bulliform cells. Map‐based cloning and MutMap analysis revealed the candidate gene *LOC_Os05g32110* (*ACL83*) encoding a COBRA‐like protein. Complementation and CRISPR/Cas9 knockout assays confirmed that *ACL83* is responsible for the mutant phenotype of *acl83*. *ACL83* was constitutively expressed, and its encoded protein was localized to the plasma membrane. Furthermore, the transcription factor ROC5 directly bound to the promoter of *ACL83* to activate *ACL83* expression. Meanwhile, the *acl83*/*roc5* double mutant exhibited an abaxially curled leaf phenotype similar to that of the *acl83* single mutant, suggesting that *ACL83* is a downstream target gene of *ROC5*. Collectively, our findings revealed a distinct ROC5‐ACL83 genetic pathway in which the COBRA‐like protein ACL83 functions downstream of ROC5 to control abaxial leaf rolling in rice by regulating cell expansion. This work provides insight into the role of COBRA‐like proteins in the regulation of leaf morphogenesis.

## Significance Statement

*ACL83* encodes a COBRA‐like protein and acts as a downstream target gene of the HD‐ZIP IV transcription factor ROC5 to control abaxial leaf rolling in rice by regulating cell expansion. This work provides insight into the role of COBRA‐like proteins in the regulation of leaf morphogenesis.

## INTRODUCTION

Rice (*Oryza sativa*) is a staple food for a large proportion of the global population (Zhang et al., 2024), and improving grain yield has long been a central objective of rice research (Liu et al., 2022; Yuan et al., 2022). Leaf functions, including photosynthesis, respiration, and transpiration, depend on leaf shape or three‐dimensional architecture (Govaerts et al., 1996). In rice, moderate leaf rolling is regarded as a desirable agronomic trait, which contributes to yield improvement by reshaping plant architecture, enhancing photosynthetic efficiency, reducing transpirational water loss, delaying leaf senescence, and optimizing canopy light interception, particularly during the later stage of plant growth (O'Toole & Cruz, 1980; Richards et al., 2002). Therefore, isolating mutants with moderately rolled leaves and identifying genes responsible for leaf rolling will facilitate the development of super‐high‐yielding rice varieties with an ideal plant architecture (Liu et al., 2016).

Bulliform cells (BCs) are widely regarded as playing a key role in the regulation of rice leaf rolling (Zou et al., 2011). These specialized large parenchymatous cells occur in groups between adjacent vascular bundles on the adaxial surface of rice leaves, and possess a large cell volume, highly vacuolated cytoplasm, and thin cell walls (Wang et al., 2020). In rice, most leaf rolling phenotypes are attributable to abnormalities in the morphology, number, size, or spatial distribution of BCs on the adaxial surface of the leaf blade (Li, Zhang, et al., 2017). Nevertheless, it was found in some studies that sclerenchyma cells, epidermal cells, mesophyll cells, and cuticular wax all contribute to the regulation of leaf rolling. For example, *SLL1* (*shallot‐like 1*) promotes the development of abaxial sclerenchyma cells in leaves, and its mutation results in defective sclerenchyma formation on the abaxial side and consequently extremely incurved leaves (Zhang et al., 2009). *CFL1* (*curly flag leaf 1*) negatively regulates leaf cuticle development, thereby altering leaf morphology (Wu et al., 2011). *OsCHR4* regulates cuticle wax formation, and its mutation results in narrower and rolled leaves with increased cuticular wax (Guo et al., 2019). In addition, several genes involved in secondary cell wall or cellulose biosynthesis also play important roles in determining leaf rolling, such as *NRL1* (*narrow and rolled leaf 1*)/*DNL1* (*dwarf and narrowed leaf 1*), *RL14* (*rolling‐leaf 14*), *SRL1* (*semi‐rolled leaf 1*)*/CLD1* (*curled leaf and dwarf 1*), *OsMYB103L* (R2R3‐MYB transcription factor), *OsSND2* (NAC family transcription factor), and *PSL1* (*photo‐sensitive leaf rolling 1*) (Ding et al., 2015; Fang et al., 2012; Hu et al., 2010; Li, Zhang, et al., 2017; Xiang et al., 2012; Yang et al., 2014; Ye et al., 2018; Zhang et al., 2021).

Based on the direction of rolling, rice leaf rolling is generally classified into two types: adaxial rolling and abaxial rolling. So far, many genes have been identified to modulate adaxially curled leaves, such as *OsAGO7* (*Argonaute family*), *REL1* (*rolled and erect leaf 1*), and *REL2*, *OsLBD3‐7* (*lateral organ boundaries domain*), *OsHox32* (*HD‐ZIP III family member*), *SRL2* (*semi‐rolled leaf2*), *NRL2* (*narrow and rolled leaf2*), *LRRK1* (*leaf rolling receptor‐like cytoplasmic kinase 1*), *OsDRB2* (*double‐stranded RNA‐binding2*)/*SRL10* (*semi‐rolled leaf 10*), *OsLC1* (leaf‐curling mutant) (Chen et al., 2015; Li, Shen, et al., 2016; Li, Zou, et al., 2016; Liu et al., 2016; Shi et al., 2007; Wang et al., 2023; Wu et al., 2025; Yang et al., 2016; Yuan et al., 2022; Zhao et al., 2016; Zhou et al., 2018). Meanwhile, seven genes encoding transcription factors have been found to regulate abaxially curled leaves, including *ACL1* (*abaxially curled leaf1*) and *ACL2*, *OsZHD1* and *OsZHD2*, *ROC1* (*Rice Outermost Cell‐specific gene1*), *ROC5*, and *ROC8*/*Url1* (*upward rolled leaf 1*). *ACL1* and *ACL2* encode HD‐ZIP III transcription factors, *OsZHD1* and *OsZHD2* encode a zinc finger homeodomain class homeobox transcription factor, and *ROC1*, *ROC5*, and *ROC8*/*Url1* encode homeodomain‐leucine zipper (HD‐ZIP IV) family transcription factors. Overexpression of *ACL1*, *ACL2*, *OsZHD1*, and *OsZHD2* leads to abaxially curled leaves (Li et al., 2010; Xu et al., 2014). In contrast, overexpression of *ROC1*, *ROC5*, or *ROC8* causes adaxially curled leaves, whereas their knockout mutants all show abaxially curled leaves (Fang et al., 2021; Sun et al., 2020; Tao et al., 2024; Xu et al., 2021; Zou et al., 2011). Nevertheless, the involvement of non‐transcription factor genes in abaxial leaf rolling remains poorly understood.

COBRA encodes a glycosylphosphatidylinositol (GPI)‐anchored protein and belongs to a multigene family (COBRA‐like family) in plants (Roudier et al., 2002). In rice, COBRA‐like proteins primarily function in the mechanical strength of tissues/organs and/or cell wall biosynthesis (Dai et al., 2011; Li et al., 2003), and have also been implicated in the regulation of β‐glucan levels in the endosperm cell wall and in stomatal development (Li et al., 2021; Midorikawa et al., 2019). However, their roles in the regulation of leaf rolling remain unclear.

In this study, we isolated a leaf‐rolling mutant, *acl83*, from *indica* rice, which exhibited an abaxially curled leaf phenotype due to increased area of BCs. Map‐based cloning and MutMap analysis suggested that the candidate gene *ACL83* encodes a COBRA‐like protein. Complementation and CRISPR/Cas9 knockout assays confirmed that *ACL83* is responsible for the mutant phenotype of *acl83*. This gene was constitutively expressed, and protein ACL83 was localized to the plasma membrane. Furthermore, our data demonstrated that *ACL83* is a downstream target gene of the HD‐ZIP IV transcription factor ROC5. Therefore, our findings revealed a distinct ROC5‐ACL83 genetic pathway in which the COBRA‐like protein ACL83 functions downstream of ROC5 to control abaxial leaf rolling in rice by regulating cell expansion.

## RESULTS

### Phenotypic characterization of the *acl83* mutant

We isolated the *acl83* mutant from an ethyl methane sulfonate (EMS) mutagenesis library of *indica* rice restorer line 676R. The mutant did not exhibit a leaf‐rolling phenotype during early growth. However, from the late tillering stage onward, *acl83* developed an abaxially curled leaf phenotype that persisted until maturity, with the flag leaf showing a milder rolling phenotype than the lower leaves (Figure 1A–C). At the grain‐filling stage, the leaf rolling indices (LRIs) of the flag leaf, second, and third leaves in *acl83* plants were 35.4, 70.0, and 64.6%, respectively, whereas the corresponding wild‐type leaves showed slight adaxial rolling with LRI values of 11.9, 9.0, and 8.6%, respectively (Figure 1D). At maturity, the investigation of agronomic traits showed that plant height, number of productive panicles per plant, and number of spikelets per panicle of *acl83* were significantly reduced by 19.7, 25.6, and 18.7%, respectively, compared with those of the wild type (WT) (Figure 1E–G). In contrast, seed setting rate and 1000‐grain weight showed no significant differences between *acl83* and WT (Figure 1H,I).

**Figure 1:**
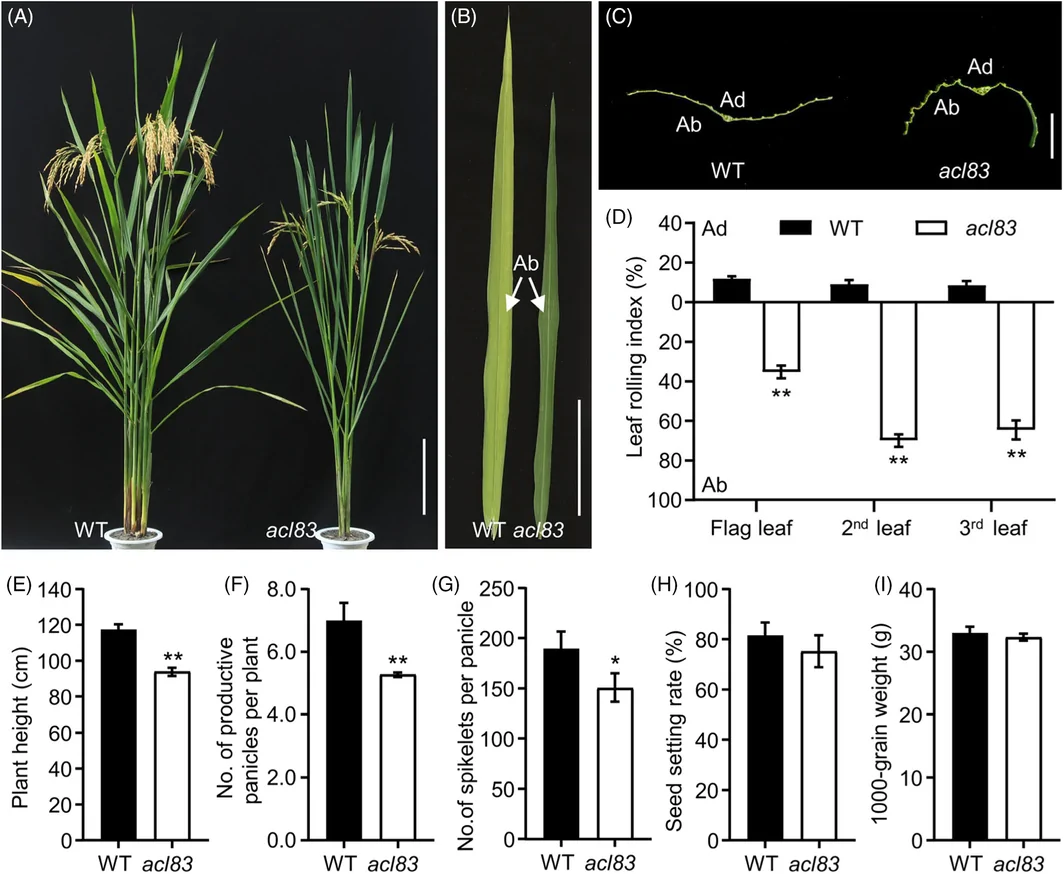
Phenotypes characterization of the *acl83* mutant. (A) Plant phenotypes of *acl83* and its wild type (WT) at grain‐filling stage. Scale bar, 20 cm. (B, C) Blade phenotypes and transverse sections of the second leaves from the top (the top 2nd leaves) at the grain‐filling stage, respectively. Ab, abaxial; Ad, adaxial. Scale bars, 10 cm in (B) and 0.5 cm in (C). (D) Leaf rolling indices of the top three leaves (the flag, 2nd, and 3rd leaves from the top of the main stem of a single plant) at the grain‐filling stage. Ad and Ab indicate adaxial and abaxial rolling, respectively. (E–I) Plant height, number of productive panicles per plant, number of spikelets per panicle, seed setting rate, and 1000‐grain weight at the maturity stage. Values are means ± SD of three biological replicates. Asterisks indicate significant differences between *acl83* and WT by Student's *t*‐test (*P < 0.05, **P < 0.01).

To investigate the formation of leaf rolling, we performed paraffin cross‐sectioning on the middle region of the second leaf from the top (the top 2nd leaf) at the heading stage. As a result, the number of bulliform cells (BCs) in the midrib, large, and small veins did not differ significantly between *acl83* and WT (Figure 2A,B). However, the total area of BCs adjacent to the midrib, large, and small veins in *acl83* was significantly increased by 56.3, 91.7, and 110.0%, respectively, compared with that in WT (Figure 2A,C). This result suggested that the abaxial leaf rolling could be due to the increased size of BCs in the mutant.

**Figure 2:**
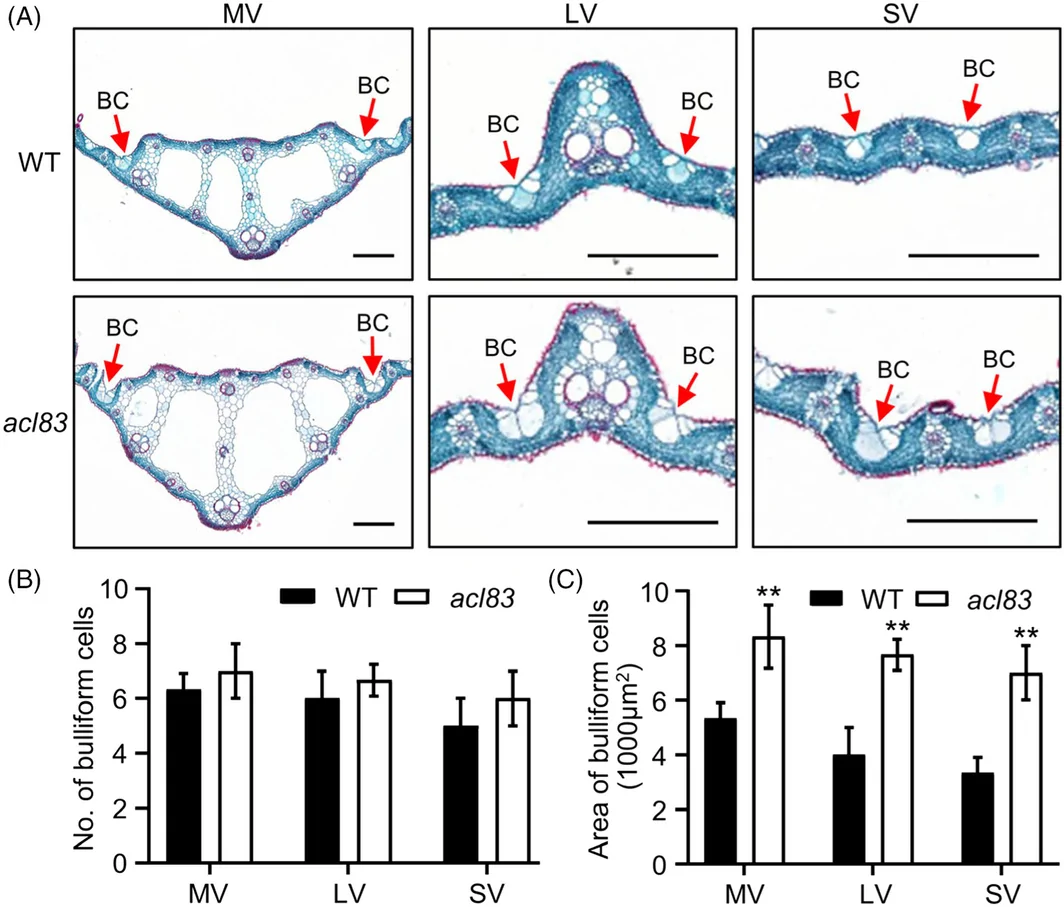
Comparison of bulliform cells between *acl83* and WT. (A) Paraffin sections of the middle region of the top 2nd leaves at the heading stage. Ab, abaxial; Ad, adaxial; LV, large vein; MV, midrib vein; SV, small vein. Red arrows indicate bulliform cells (BCs). Scale bars, 250 μm. (B, C) Number and total area of bulliform cells in the top 2nd leaves at the heading stage. Values are means ± SD of three biological replicates. Asterisks indicate significant differences between *acl83* and WT by Student's *t*‐test (**P < 0.01).

### Gene cloning of *ACL83*

For genetic analysis, we crossed *acl83* with 676R (WT) plants. All individuals in the F_1_ population exhibited a normal phenotype as WT. While the F_2_ population displayed a segregation of the two phenotypes, the segregation ratio of WT‐like to *acl83*‐like individuals demonstrated a ratio of 3:1 (*χ* ^2^ = 2.43 < $\chi_{0.05}^{2}=3.84$) (Table S1). The result suggested that the *acl83* mutant phenotype was controlled by a single recessive nuclear gene.

To identify the causal gene for *acl83*, SSR, and InDel markers, along with an F_2_ population derived from the *acl83* × ZH11 cross, were used to map the mutated locus. The initial mapping using 350 F_2_ individuals with abaxially curled leaves showed that the *acl83* locus was linked to the SSR marker RM163 on the long arm of chromosome 5 (Figure 3A). After that, 895 F_2_ plants with the mutant phenotype and eight InDel markers with polymorphism between the two parents were used to fine map the *acl83* locus (Figure 3B). The target locus was ultimately narrowed down to a 196‐kb region between InDel markers P5 and P7, at a genetic distance of 0.11 cM and 0.28 cM, respectively (Figure 3C). Within the 196‐kb region, 27 genes are annotated in the Rice Genome Annotation Project (https://rice.uga.edu) (Table S2).

**Figure 3:**
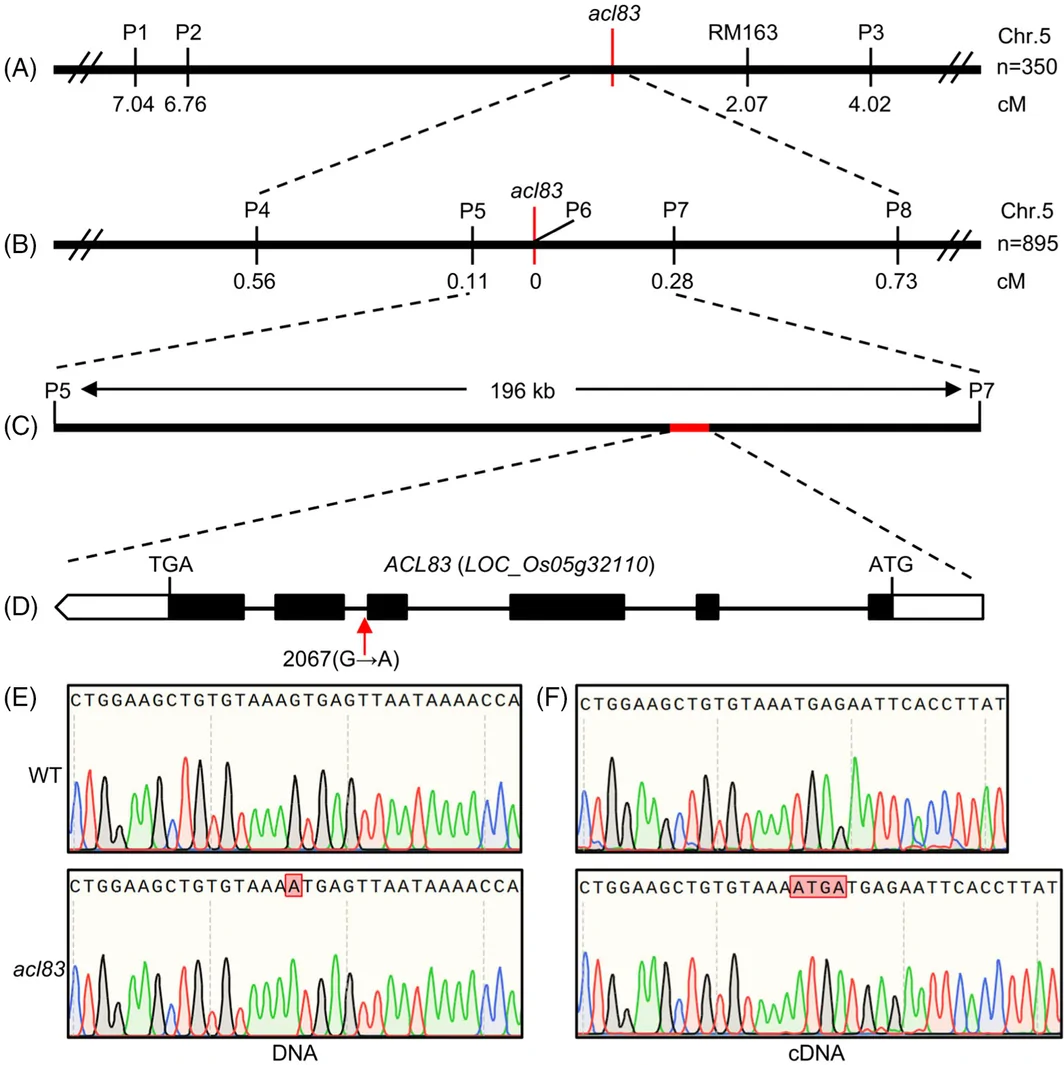
Mapping of the *acl83* locus and identification of the *acl83* mutation. (A) Initial mapping of the *acl83* locus on the long arm of chromosome 5 using 350 individuals exhibiting abaxially curled leaves from the *acl83* × ZH11 F_2_ population. (B) Fine mapping of the *acl83* locus using 895 mutant individuals, which was narrowed down to a 196‐kb region between InDel markers P5 and P7. (C) Only one gene (*LOC_Os05g32110* indicated with the red box) in the 196‐kb region was found to carry a mutation by MutMap analysis. (D) Gene structure of the candidate gene *ACL83* (*LOC_Os05g32110*). Black boxes represent exons and black lines represent introns. A red arrow denotes the single nucleotide G‐to‐A substitution occurring at position 2067, which is located at the first base of the fourth intron of *ACL83* in the *acl83* mutant. (E) Genomic DNA sequencing confirming the G‐to‐A substitution in *acl83* compared with the wild type (WT). (F) cDNA sequencing revealing an additional “ATGA” insertion in *acl83* compared with WT.

Subsequently, we performed whole‐genome resequencing and MutMap analysis using 30 F_2_ individuals with abaxially curled leaves from the *acl83* × 676R cross and found a single nonsynonymous mutation in *LOC_Os05g32110*, the only variant within the 196‐kb candidate region. We sequenced the *LOC_Os05g32110* gene in genomic DNA from the *acl83* mutant and the wild‐type parent 676R. Consistent with the MutMap analysis, a single nucleotide G to A substitution occurred at position 2067 of this gene sequence (corresponding to the first nucleotide of the fourth intron) in the *acl83* mutant (Figure 3D,E). We next sequenced cDNA of *LOC_Os05g32110* from *acl83* mutant and the wild type using reverse transcription (RT)‐PCR. The result showed that the *acl83* cDNA harbored a 4‐bp insertion (ATGA) (Figure 3F), which introduced a premature termination codon and resulted in a truncated protein. Therefore, *LOC_Os05g32110* was identified as the candidate gene responsible for the *acl83* mutant phenotype and designated *ACL83*.

Sequencing analysis revealed that *ACL83* (*LOC_Os05g32110*) comprises six exons and five introns (Figure 3D), with a genomic span of 2851 bp and a coding sequence (CDS) of 1374 bp. The gene encodes a putative GPI‐anchored COBRA‐like protein (Figure S1A) (Dai et al., 2009). Phylogenetic analysis showed that ACL83 is more closely related to its maize (*Zea mays*) homolog than to the homologs from other species (Figure S1B).

Previous studies have revealed that *LOC_Os05g32110* (*OsBC1L4*) participates in cell wall biosynthesis (Dai et al., 2011). Therefore, we analyzed the cell wall components of leaves from the *acl83* mutant at the heading stage, when the abaxial leaf rolling phenotype was clearly evident. As a result, hemicellulose and lignin contents in the *acl83* leaves were reduced by 26.3 and 22.3%, respectively, compared to the wild type (Figure S2B,C), whereas cellulose and pectin contents did not differ significantly (Figure S2A,D). These results indicate that the cell wall composition is altered in the *acl83* mutant.

### Complementation and CRISPR/Cas9 knockout of *ACL83*

To confirm that *ACL83* is the causal gene for *acl83*, we conducted a complementation experiment. The full‐length cDNA of the wild‐type *ACL83* gene was inserted into the pCAMBIA2300‐*Actin1* vector to generate a complementation construct. Because the *indica* rice variety 676R is recalcitrant to *Agrobacterium*‐mediated transformation, we generated an *acl83* near‐isogenic line (NIL) in the genetic background of the transformable *japonica* variety Zhonghua 11 (ZH11) through repeated backcrossing and selfing (Figure S3). Then, the complementation construct was introduced into the *acl83*‐NIL (NIL^ZH11^), yielding 10 positive transgenic lines that were confirmed by PCR (Figure S4A). These transgenic lines all exhibited a normal leaf phenotype indistinguishable from the wild type (ZH11), with restored leaf rolling indices and BC morphology and size (Figure 4A,B,G,I,J). In addition, the transgenic lines expressing *ACL83* fully rescued major agronomic traits, including plant height, number of productive panicles per plant, panicle length, number of spikelets per panicle, seed setting rate, and 1000‐grain weight (Figure S4B–G).

**Figure 4:**
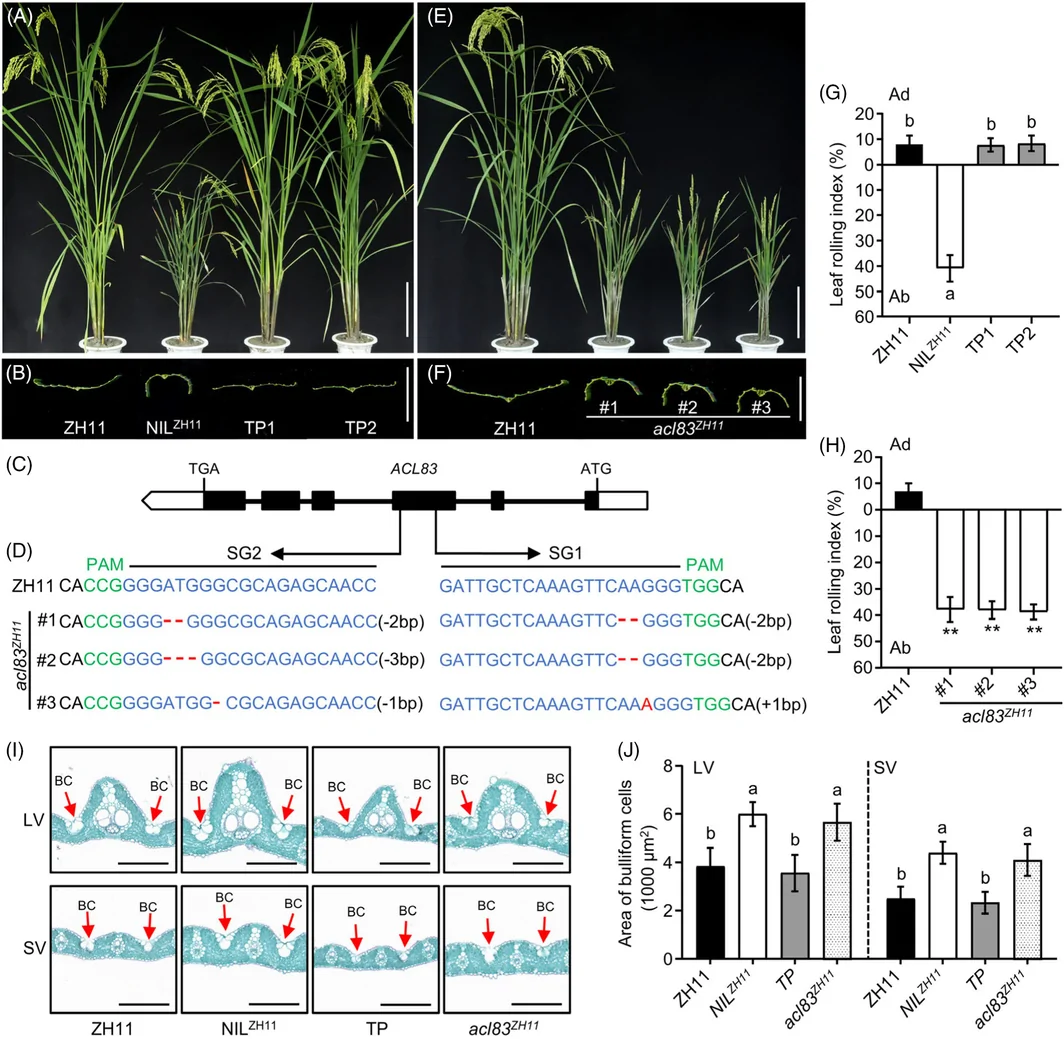
Functional validation of *ACL83* by complementation and CRISPR/Cas9‐mediated knockout. (A, B) Plant architectures of ZH11, NIL^ZH11^ and transgenic plants (TP1 and TP2) at the grain‐filling stage, and their transverse sections of the top 2nd leaves, respectively. Scale bars, 20 cm in (A) and 1 cm in (B). (C) Gene structure of *ACL83* (*LOC_Os05g32110*) and its sgRNA target sites used for CRISPR/Cas9‐mediated gene knockout. (D) Alignment of the CRISPR/Cas9 target sites in *ACL83* between the *japonica* cultivar ZH11 and the knockout lines (*acl83* ^ZH11^ #1, #2 and #3). Blue letters indicate target sequences; green letters indicate the protospacer adjacent motif (PAM) sites; red dashes and letters indicate nucleotide deletions and insertions, respectively. (E, F) Plant architectures of ZH11 and the knockout lines at the heading stage, and their transverse sections of the top 2nd leaves, respectively. Scale bars, 20 cm in (E) and 0.5 cm in (F). (G, H) Leaf rolling indices of the top 2nd leaves in ZH11, NIL^ZH11^, TP and knockout lines at the grain‐filling stage. Ad and Ab indicate adaxial and abaxial rolling respectively. (I, J) Paraffin sections and area quantification of bulliform cells, respectively, in the top 2nd leaves in ZH11, NIL^ZH11^, TP and *acl83* ^*ZH11*^ at the grain‐filling stage. LV, large vein; SV, small vein. Red arrows indicate bulliform cells (BCs). Scale bar, 200 μm. Values are means ± SD of three biological replicates. In panels (G) and (J), statistical significance was determined by one‐way ANOVA with Tukey's multiple‐comparison test (*P* < 0.05), and different lowercase letters indicate significant differences. In panel (H), asterisks indicate significant differences from ZH11 as determined by Student's *t*‐test (**P < 0.01).

To further validate the function of *ACL83*, we used the CRISPR/Cas9 system to generate knockout lines (*acl83* ^*ZH11*^) in the ZH11 background. Sequence analysis confirmed that three knockout lines harbored insertions or deletions at both sgRNA target sites SG1 and SG2 (Figure 4C,D; Figure S5A), resulting in predicted premature termination of the ACL83 protein (Figure S5B). Importantly, the three homozygous knockout lines all exhibited an abaxially curled leaf phenotype similar to that of the *acl83*‐NIL (NIL^ZH11^) (Figure 4E,F). The leaf rolling indices of the top 2nd leaves were 37.9%, 38.1%, and 38.8% in the three lines, respectively, whereas the wild‐type ZH11 leaf showed only slight adaxial rolling (LRI = 6.9%) (Figure 4H). Meanwhile, the total area of BCs adjacent to large veins and small veins was significantly increased in the knockout lines by 47.8 and 64.0%, respectively, compared to that in the wild‐type ZH11 (Figure 4I,J). In addition, the major agronomic traits were significantly impaired in the knockout lines (Figure S6A–F).

Finally, we generated *ACL83*‐overexpressing lines in the ZH11 background to assess the effect of elevated *ACL83* expression. qRT‐PCR analysis confirmed that *ACL83* transcript levels were markedly upregulated in the overexpression lines (Figure S7A). Phenotypic observations revealed no significant differences in leaf morphology or leaf rolling index between the overexpression lines and the wild‐type ZH11 plants (Figure S7B–D).

Collectively, both complementation and knockout assays demonstrated that *ACL83* is essential for leaf morphology development in rice, and that a single‐nucleotide mutation in this gene causes the abaxially curled leaf phenotype of the *acl83* mutant.

### Expression pattern and subcellular localization

To explore the expression pattern of *ACL83*, we quantified its transcript levels in various tissues of wild‐type 676R plants at both the seedling and booting stages by qRT‐PCR. *ACL83* was expressed in all tissues examined, including roots, stems, leaf blades, leaf sheaths, and young panicles, although the expression levels varied among various organs. Notably, the highest expression at the booting stage was observed in stems and leaf blades (Figure 5A). We further analyzed *ACL83* expression in leaf blades during different developmental stages and found that transcript levels gradually increased from the 10th to the 15th leaf, with peak expression detected in the 15th leaf (the flag leaf) (Figure 5B).

**Figure 5:**
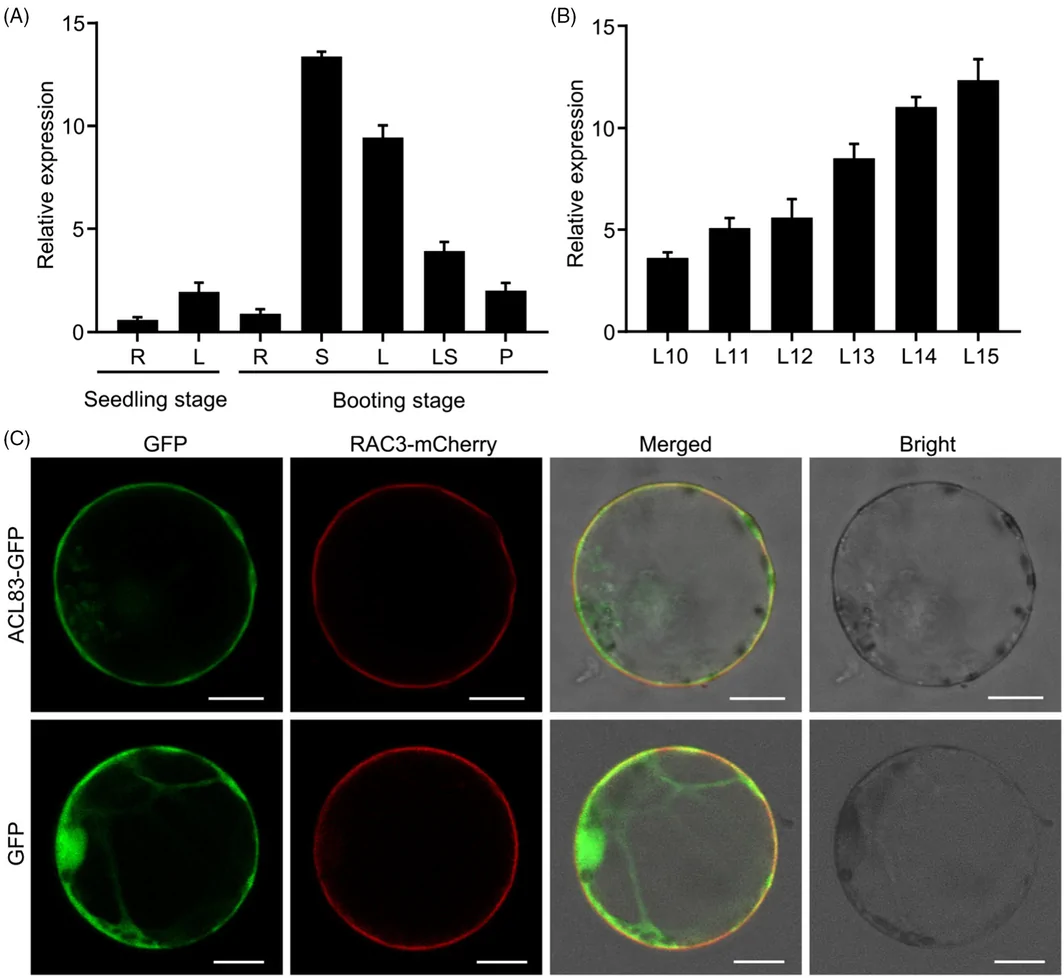
Expression pattern of the *ACL83* gene and subcellular localization of the ACL83 protein. (A) The spatiotemporal expression of *ACL83* in root (R), leaf blade (L), stem (S), leaf sheath (LS), and young panicle (P) of the wild‐type plants at seedling stage and booting stage. (B) Expression analysis of *ACL83* in the wild‐type leaf blades at different developmental stages. L10, L11, L12, L13, L14, and L15 indicate the full‐expanded leaves from the stem base, respectively (L15 was the flag leaf). Values are means ± SD of three biological replicates. (C) Subcellular localization of ACL83 protein. The fusion protein ACL83‐GFP and the empty GFP were co‐expressed with the plasma membrane marker RAC3‐mCherry, respectively. Green fluorescence indicates the signals from GFP protein, red fluorescence indicates the signals from mCherry protein, and yellow fluorescence shows images with two types of fluorescence merged. Scale bars, 10 μm.

To investigate subcellular localization of the ACL83 protein, an ACL83‐GFP recombinant vector was constructed and co‐expressed with the plasma membrane marker RAC3‐mCherry in rice protoplasts. As a result, the GFP signals from the ACL83‐GFP fusion protein precisely overlapped with the red fluorescence of the plasma membrane marker RAC3‐mCherry, whereas the green fluorescence from the GFP control showed no overlap with the red fluorescence of RAC3‐mCherry (Figure 5C). These results indicated that ACL83 is localized to the plasma membrane.

### ROC5 activates the promoter of *ACL83* via direct binding to the L1‐box motif

The leaf rolling phenotype of the *acl83* mutant was similar to that of the *roc5* mutant (Zou et al., 2011). Given that ROC5 is a HD‐ZIP IV transcription factor, we hypothesized that ROC5 might act upstream of *ACL83* in a common genetic pathway. To test this, we performed a yeast one‐hybrid (Y1H) assay, which showed that ROC5 could bind to the *ACL83* promoter (the 2071‐bp region upstream of the ATG) and activate the *LacZ* reporter gene (Figure 6A). Subsequently, we conducted a dual‐luciferase (LUC) assay. The firefly luciferase reporter (*ACL83pro*::*LUC*) was driven by the *ACL83* promoter, with 35S‐driven *Renilla* luciferase (*REN*) serving as an internal control. The reporter construct was co‐transformed into tobacco leaf epidermal cells with either pC2300‐CaMV35S‐*ROC5* or pC2300‐CaMV35S‐*GFP* (negative control) (Figure 6B). The results showed that the LUC fluorescence signal was markedly higher in the leaf cells co‐expressing ROC5 and *ACL83pro*::*LUC* than in those co‐expressing GFP and *ACL83pro*::*LUC* (control) (Figure 6C). Quantification of LUC and REN activities revealed that the LUC/REN ratio in the ROC5 effector group was 4.6‐fold higher than that in the GFP control (Figure 6D). A similar trend was observed in rice protoplasts (Figure S8).

**Figure 6:**
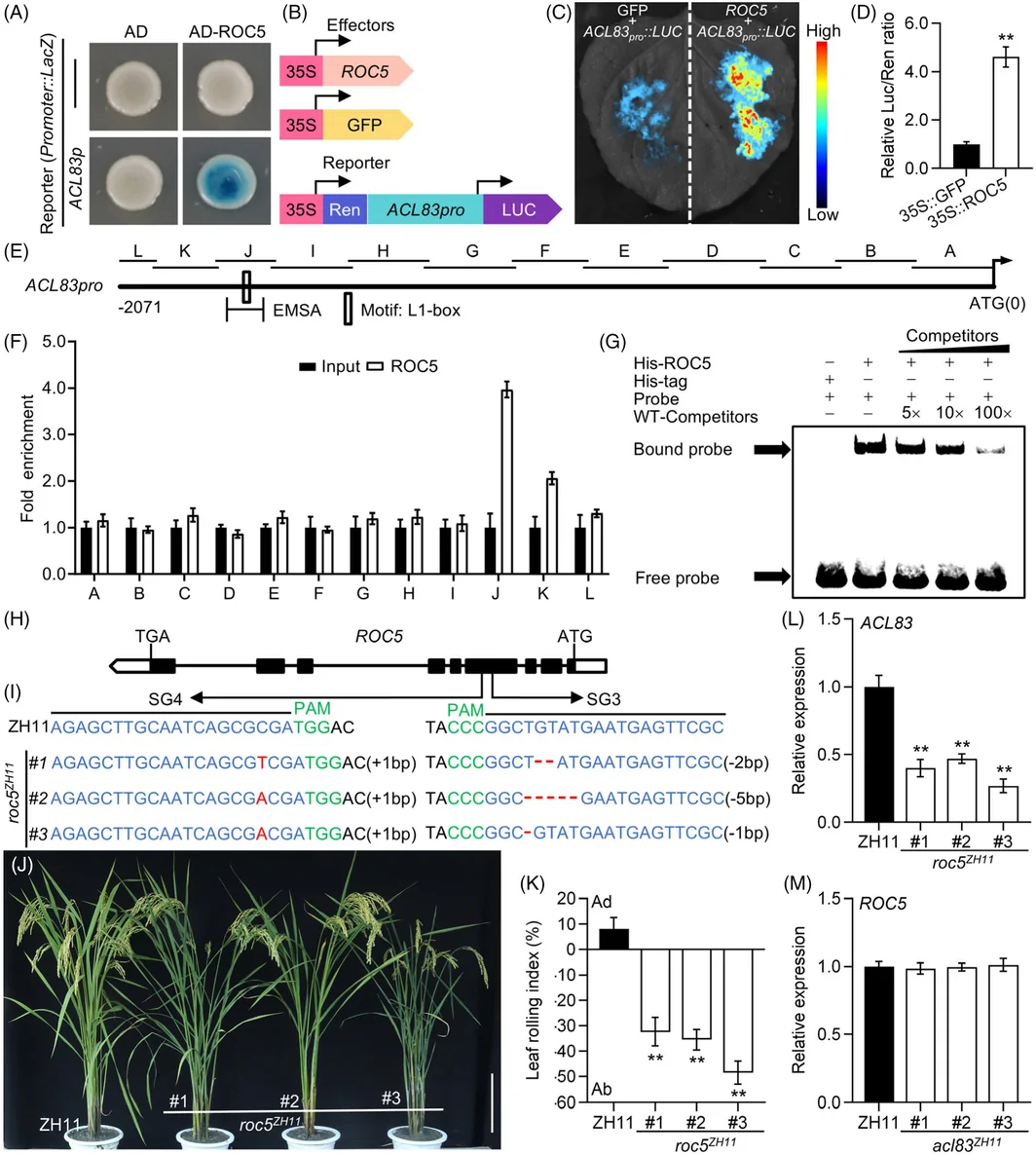
ROC5 directly regulates the target gene *ACL83*. (A) Yeast one‐hybrid assay showing that AD‐ROC5 activates the *ACL83* promoter‐driven *LacZ* reporter. (B) Schematic representation of the effector and reporter constructs used for the dual‐luciferase assay. LUC, firefly luciferase; REN, Renilla luciferase. (C) Chemiluminescence imaging of *Nicotiana benthamiana* leaves co‐infiltrated with *ACL83pro*::LUC together with GFP (left) or ROC5 (right). (D) Activation of the *ACL83* promoter by *ROC5* in *N. benthamiana* leaves. The LUC/REN ratio of the GFP control was set to 1. (E) Schematic diagram of the *ACL83* promoter divided into 12 fragments (A–L) for DAP‐qPCR analysis. DNA sequences containing the L1‐box motif are indicated by white boxes. (F) DAP‐qPCR analysis of *ROC5* binding to *ACL83* promoter fragments. Input DNA fragments without DAP incubation were used as a negative control and normalized to 1. (G) Electrophoretic mobility shift assay (EMSA) showing binding of His‐*ROC5* to the L1‐box‐containing probe in the *ACL83* promoter. Increasing amounts (5×, 10× and 100×) of unlabeled wild‐type competitor probe were added as indicated. (H) Gene structure of *ROC5* and design of CRISPR/Cas9 target sites. (I) Sequence alignment of the CRISPR/Cas9 target sites in *ROC5* between the *japonica* cultivar ZH11 and the *ROC5*‐knockout lines (*roc5* ^*ZH11*^ #1, #2 and #3). Blue letters indicate target sequences; green letters indicate PAM sites; red dashes and letters indicate nucleotide deletions and insertions, respectively. (J) Plant architectures of ZH11 and the *ROC5*‐knockout lines at the grain‐filling stage. Scale bar, 20 cm. (K) Leaf rolling indices of the top 2nd leaves in ZH11 and the *ROC5*‐knockout lines at the grain‐filling stage. Ad and Ab indicate adaxial and abaxial rolling, respectively. (L, M) Relative expression levels of *ACL83* in *ROC5*‐knockout lines and *ROC5* in *ACL83*‐knockout lines, respectively. Total RNA was extracted from leaves at the booting stage, and relative expression levels were normalized to *Actin1*. In (L), the expression level of *ACL83* in ZH11 was set to 1.0, and values in the *ROC5*‐knockout lines were calculated accordingly. In (M), the expression level of *ROC5* in ZH11 was set to 1.0, and values in the *ACL83*‐knockout lines were calculated accordingly. Values are means ± SD of three biological replicates. Asterisks indicate significant differences from ZH11 as determined by Student's *t*‐test (**P < 0.01).

To further confirm the direct binding of ROC5 to the *ACL83* promoter, we expressed and purified recombinant ROC5 protein (Figure S9), and then performed DNA affinity purification followed by qPCR (DAP‐qPCR). The result suggested that promoter fragments J and K were enriched 6.0‐fold and 1.0‐fold, respectively, relative to the control (Figure 6E,F), indicating that *ROC5* directly binds to fragment J of the *ACL83* promoter *in vitro*. HD‐ZIP IV transcription factor regulates downstream target genes by binding to the L1‐box motif (5′‐TAAATGYA‐3′) (Abe et al., 2003; Fang et al., 2021; Wu et al., 2011). Notably, this motif is present in fragment J of the *ACL83* promoter. Therefore, we performed an electrophoretic mobility shift assay (EMSA) to test whether ROC5 directly binds to a probe containing the L1‐box sequence. The results showed that His‐ROC5, but not the His tag alone, specifically bound to the L1‐box motif within fragment J of the *ACL83* promoter (Figure 6G).

Taken together, our *in vivo* and *in vitro* results demonstrated that ROC5 positively regulates *ACL83* by directly binding to its promoter, suggesting that *ACL83* could act downstream of *ROC5* to regulate leaf development.

### *ACL83* acts downstream of ROC5 in a genetic pathway regulating abaxial leaf rolling

To confirm the genetic relationship between *ACL83* and *ROC5*, we used the CRISPR/Cas9 system to generate a *roc5* single‐knockout mutant by targeting *ROC5* alone (Figure 6H), and an *acl83/roc5* double‐knockout mutant by simultaneously targeting both *ACL83* and *ROC5* (Figure 7A), all in the ZH11 genetic background.

**Figure 7:**
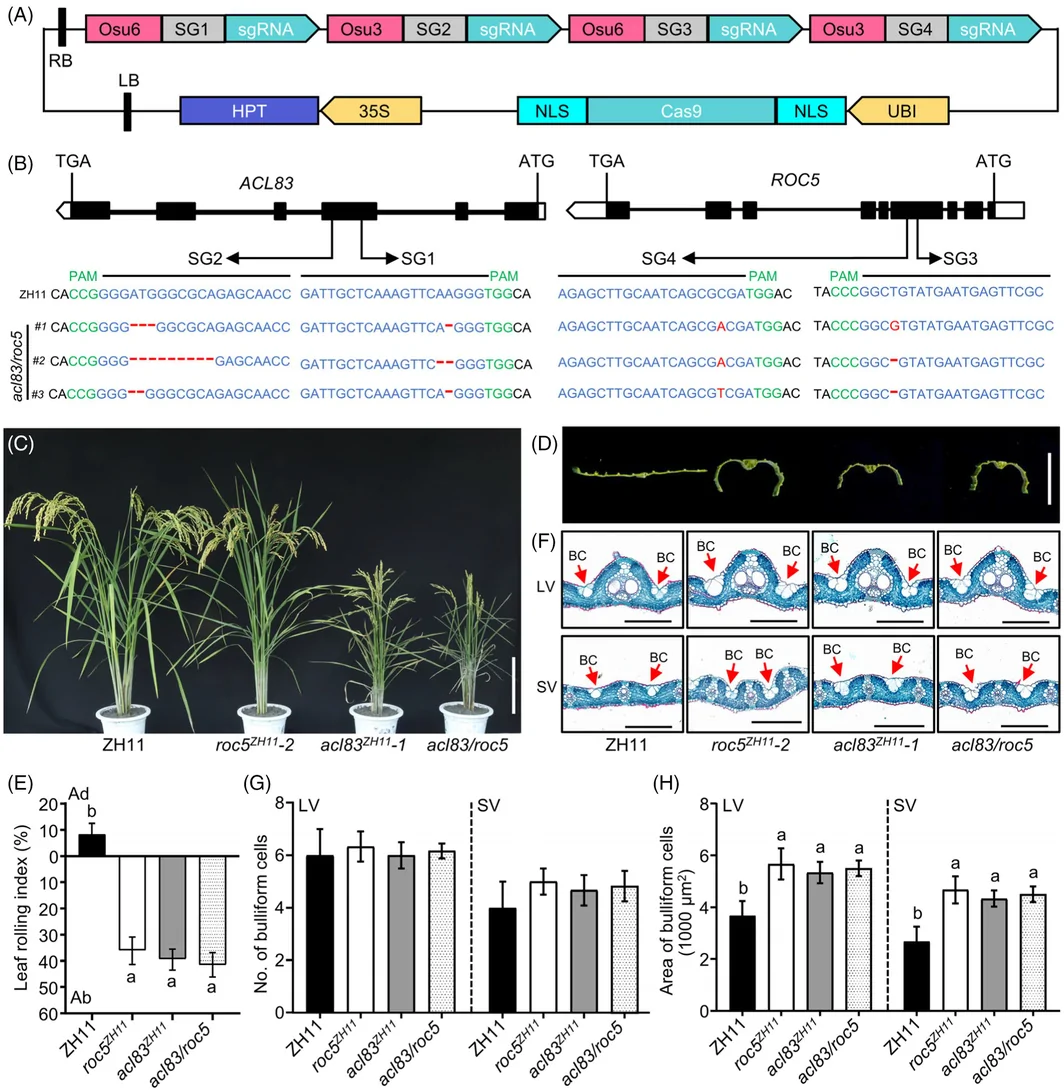
Genetic evidence showing that *ACL83* acts downstream of *ROC5* to regulate bulliform cell development and leaf rolling. (A) Schematic representation of the CRISPR/Cas9 construct containing four sgRNA expression cassettes (SG1 and SG2 targeting *ACL83*, and SG3 and SG4 targeting *ROC5*). (B) Gene structures of *ACL83* and *ROC5*, and their sequence alignment of the CRISPR/Cas9 target sites between the ZH11 and the *acl83/roc5* double‐mutant lines (#1, #2, and #3). Blue letters indicate target sequences; green letters indicate PAM sites; red dashes and letters indicate nucleotide deletions and insertions, respectively. (C, D) Plant architectures of ZH11, *roc5* ^*ZH11*^ *‐2*, *acl8* ^*3ZH11*^ *‐1*, and the double mutant *acl83/roc5*, and their transverse sections of the top 2nd leaves at the grain‐filling stage. Scale bars, 20 cm in (C) and 0.5 cm in (D). (E) Leaf rolling indices of the top 2nd leaves in ZH11, *roc5* ^*ZH11*^, *acl83* ^*ZH11*^, and *acl83/roc5* at the grain‐filling stage. Ad and Ab indicate adaxial and abaxial rolling respectively. (F) Paraffin sections of the leaves in ZH11, *roc5* ^*ZH11*^ *‐2*, *acl83* ^*ZH11*^ *‐1*, and *acl83/roc5* at the heading stage. LV, large vein; SV, small vein. Red arrows indicate bulliform cells (BCs). Scale bars, 200 μm. (G, H) Number and total area of bulliform cells in the top 2nd leaves from ZH11, *roc5* ^*ZH11*^, *acl83* ^*ZH11*^, and *acl83/roc5*. LV, large vein; SV, small vein. Values are means ± SD of three biological replicates. Statistical significance was determined by one‐way ANOVA with Tukey's multiple‐comparison test (*P* < 0.05), and different lowercase letters indicate significant differences.

In the *roc5* single‐knockout mutant, three independent lines harbored insertions or deletions at two CRISPR/Cas9 target sites SG3 and SG4 (Figure 6I; Figure S10A), resulting in premature translational termination and a truncated ROC5 protein (Figure S10B). As expected, all three lines exhibited a markedly abaxially curled leaf phenotype (Figure 6J), with leaf rolling indices of 32.4, 35.5, and 48.5%, respectively (Figure 6K). qRT‐PCR analysis revealed that *ACL83* expression was significantly reduced in these *roc5* knockout lines (Figure 6L). In contrast, *ROC5* expression was unchanged in the three independent *acl83* knockout lines (Figure 6M).

In the *acl83/roc5* double‐knockout mutant, three homozygous lines harbored deletions or insertions at all four CRISPR/Cas9 target sites (SG1 to SG4) in both *ACL83* and *ROC5* (Figure 7B; Figure S11A,B), resulting in truncated ACL83 and ROC5 proteins (Figure S11C,D). Compared with wild‐type ZH11, all three *acl83/roc5* double‐knockout lines exhibited a markedly abaxially curled leaf phenotype similar to those of the *acl83* and *roc5* single‐knockout mutants (Figure 7C,D). Leaf rolling indices of the *roc5*, *acl83*, and *acl83/roc5* mutants were 36.2, 39.6, and 41.6%, respectively (Figure 7E), with no significant difference among them. Histological analysis showed that the number of BCs in the top 2nd leaves from all three mutants (*roc5*, *acl83*, and *acl83/roc5*) was comparable to that in ZH11 (Figure 7F,G), whereas the total area of BCs was markedly enlarged (Figure 7F,H). Specifically, in large veins, BC area increased by 54.5, 45.5, and 50.0% in the *roc5*, *acl83*, and *acl83 roc5* mutants, respectively, with no significant difference among them. A similar trend was observed in small veins, with increases of 75.0, 62.5, and 68.8%, respectively. Additionally, the *acl83/roc5* double mutant displayed agronomic traits similar to those of the *acl83* single‐knockout mutant, including plant height, number of productive panicles per plant, panicle length, number of spikelets per panicle, and 1000‐grain weight (Figure S12A–F).

Collectively, our data confirmed that *ACL83* acts downstream of *ROC5* in a genetic pathway to control abaxial leaf rolling in rice by regulating cell expansion.

## DISCUSSION

Leaf rolling is an important agronomic trait in rice. To date, many genes involved in the regulation of adaxially curled leaves have been cloned and functionally characterized, such as *SLL1*, *RL14*, *CLD1*/*SRL1*, *OsMYB103L*, *SRL2*, *NRL2*, *LRRK1*, *PSL1*, *DRB2/SRL10*, and *OsLC1* (Fang et al., 2012; Li, Zhang, et al., 2017; Liu et al., 2016; Wang et al., 2023; Wu et al., 2025; Xiang et al., 2012; Yang et al., 2014; Yuan et al., 2022; Zhang et al., 2009, 2021; Zhao et al., 2016; Zhou et al., 2018). However, only a few genes associated with abaxially curled leaves have been identified, including *ACL1*, *ACL2*, *OsZHD1*, *OsZHD2, ROC1*, *ROC5*, and *ROC8*/*Url1* (Fang et al., 2021; Li et al., 2010; Sun et al., 2020; Tao et al., 2024; Xu et al., 2014, 2021; Zou et al., 2011). Notably, all these genes encode transcription factors. Overexpression of *ACL1*, *ACL2*, *OsZHD1*, or *OsZHD2* leads to abaxially curled leaves. In contrast, overexpression of *ROC1*, *ROC5*, or *ROC8* causes an adaxially curled leaf phenotype, whereas their loss‐of‐function results in abaxially curled leaves. In this study, we isolated mutant *acl83*, which showed an abaxially curled leaf phenotype due to increased area of bulliform cells (BCs). The candidate gene for *acl83* is *LOC_Os05g32110* (*ACL83*) encoding a putative COBRA family protein. In the *acl83* mutant, the mutation causes aberrant splicing, leading to the premature translation termination. Complementation and knockout assays confirmed that *ACL83* is the causal gene for the *acl83* mutant phenotype. Therefore, our data indicated that *ACL83* is a distinct gene controlling abaxial leaf rolling in rice.

COBRA belongs to a plant‐specific multigene family (Roudier et al., 2002). This family comprises 12 members in Arabidopsis (*COBRA* and *AtCOBL1‐11*), 9 in maize (*ZmBK2*, *ZmBK2L1*, and *ZmBK2L3‐9*), and 11 in rice (*BC1*, *OsBC1Lp1*, and *OsBC1L1‐9*) (Dai et al., 2009). In rice, *BC1* regulates the mechanical strength of tissues/organs (Li et al., 2003); *OsBC1L1* and *OsBC1L8* are involved in regulating stomatal development (Li et al., 2021); *OsBC1L6* modulates β‐glucan levels in the endosperm cell wall (Midorikawa et al., 2019); and *OsBC1L4* affects cellulose synthesis in cell walls, with decreased cellulose content and increased pectin content in the mature internodes of the *osbc1l4* mutant (Dai et al., 2011). Nevertheless, the functional role of COBRA‐like proteins in the regulation of leaf rolling remains unclear. In our study, we demonstrated that *acl83* is allelic to *osbc1l4*, and that ACL83 localizes to the plasma membrane in rice protoplasts (Figure 5C). In the *acl83* leaves at the heading stage, hemicellulose and lignin contents were reduced, whereas cellulose and pectin contents did not differ significantly (Figure S2). We speculate that the observed differences in cell wall components between *acl83* and *osbc1l4* may result from variations in the developmental stages and tissue types sampled. Importantly, the number of BCs remained unchanged in *acl83*, whereas their area was markedly enlarged (Figure 2), supporting a role for COBRA‐like proteins in regulating cell expansion. Thus, ACL83 is a COBRA‐like protein that controls abaxial leaf rolling in rice via the regulation of cell expansion. Nevertheless, whether there is a direct causal relationship between cell wall composition changes and bulliform cell expansion remains to be determined and requires further investigation.

*ROC5* encodes a HD‐ZIP IV transcription factor in rice. *ROC5*‐knockout leads to abaxial leaf rolling by increasing the number and size of BCs on the adaxial surface, whereas *ROC5* overexpression causes adaxial leaf rolling by reducing the number and size of BCs (Xu et al., 2021; Zou et al., 2011). In addition, *ROC5* participates in a transcriptional regulatory network that coordinates multiple downstream genes to modulate BC development and leaf rolling. For example, *ROC5* regulates the epidermis‐specific gene *PFL* (*protodermal factor like*), and silencing *PFL* (via PFL‐RNAi) causes abaxial leaf rolling by increasing the number and size of BCs (Zou et al., 2011). *ROC5* directly activates *CFL2*, a cytochrome P450 gene whose mutation leads to abnormal epidermal development, enlarged BCs, and curling at the base of the flag leaf (Zhang et al., 2020). Additionally, ROC5 can form a transcriptional repressor complex with URL1 (ROC8) and TPL2 to negatively regulate *ACL1*, thereby modulating BC development and leaf rolling (Fang et al., 2021). In this study, we confirmed that ROC5 binds to the *ACL83* promoter and activates *ACL83* expression using yeast one‐hybrid and dual‐luciferase (LUC) assays (Figure 6A–D; Figure S8A). DAP‐qPCR demonstrated significant enrichment of a specific *ACL83* promoter fragment by *ROC5* (Figure 6E,F), and EMSA revealed that *ROC5* directly binds to an L1‐box motif within this fragment (Figure 6G). Furthermore, we generated *roc5* single‐knockout mutants in the ZH11 background, which exhibited an abaxially curled leaf phenotype (Figure 6J,K) and an enlarged BC area (Figure 7F–H)—phenotypes similar to those of the *acl83* single‐knockout mutant. *ACL83* transcript levels were significantly reduced in *ROC5*‐knockout lines (Figure 6L), whereas *ROC5* expression was unchanged in the *ACL83‐*knockout lines (Figure 6M). Importantly, the leaf rolling index and BC area in the *acl83*/*roc5* double mutant are not significantly greater than those in either single mutant (Figure 7E,F,H), and major agronomic traits of the double mutant largely resemble those of the *acl83* single‐knockout mutant (Figure S12A–F). Taken together, the molecular and genetic evidence support a model in which the absence of additive or synergistic effects in the *acl83*/*roc5* double mutant suggests that *ACL83* and *ROC5* act in the same genetic pathway, and the direct activation of *ACL83* by *ROC5* and the reduced expression of *ACL83* in the *roc5* single‐knockout mutant reveals that *ACL83* functions downstream of ROC5. In wild‐type plants, ROC5 activates *ACL83*, forming a ROC5‐ACL83 regulatory module that restricts BC expansion and helps maintain the balance between the adaxial and abaxial sides of the leaf blade, thereby limiting abaxial leaf rolling. When *ROC5* or *ACL83* is disrupted, reduced *ACL83* activity permits excessive BC expansion, leading to abaxial leaf rolling. In a word, our findings reveal a distinct ROC5‐ACL83 genetic pathway in which the COBRA‐like protein ACL83 functions downstream of ROC5 to control abaxial leaf rolling in rice by regulating cell expansion.

Notably, *ACL83* expression was not completely abolished in *roc5* knockout mutants (Figure 6L), suggesting that *ACL83* is not exclusively regulated by ROC5. Instead, functional redundancy among other members of the HD‐ZIP IV transcription factor family, or regulation by uncharacterized transcription factors, likely sustains basal *ACL83* expression.

## EXPERIMENTAL PROCEDURES

### Plant materials and growth conditions

The *acl83* mutant was obtained through screening an EMS mutagenesis library constructed in the *indica* rice restorer line 676R. For genetic and MutMap analyses, the mutant was crossed with the wild‐type 676R. To perform gene mapping, *acl83* was crossed with the *japonica* rice variety Zhonghua 11 (ZH11). Rice materials were grown during the normal rice growing season in the experimental fields of Sichuan Agricultural University in Chengdu, China. All the primers used in this study were listed in Table S3.

### Measurement of Leaf rolling index (LRI)

The adaxial (Ad) and abaxial (Ab) leaf rolling indices (LRIs) were measured as previously described (Shi et al., 2007). LRI value was calculated using the formula: LRI (%) = ([Lw − Ln] ÷ Lw) × 100, where Ln denotes the leaf width under the natural (folded) condition, and Lw denotes the maximum leaf width at the same position when fully unfolded.

### Histology analysis

For paraffin section analysis, leaf samples were collected from the middle region of the second leaf from the top of each plant at the heading stage, unless otherwise stated. The leaf samples were immediately submerged in 70% FAA solution (70% ethanol, 5% formaldehyde, 5% glacial acetic acid) (G1103, Servicebio, China) and fixed for 24 h, and then processed for paraffin embedding and sectioning by Servicebio (Wuhan, China). The sections were imaged and analyzed using SlideViewer software (3DHIESTECH, Hungary). Bulliform cells adjacent to the midrib, large, and small veins in the leaf samples were counted, and their total areas were measured using ImageJ software (http://rsb.info.nih.gov/ij/).

### Cell wall composition analysis

At the heading stage, flag leaves were collected from wild‐type 676R and *acl83* mutant plants, immediately frozen in liquid nitrogen, and stored at −80°C until further analysis. For cell wall composition analysis, the samples were dried at 105°C for 1 h and then at 65°C for 24 h and subsequently ground into fine powder. The contents of cellulose, hemicellulose, lignin, and pectin were determined as previously described (Dai et al., 2011).

### Gene mapping

To identify the candidate gene of *acl83*, map‐based cloning was performed using molecular markers and an F_2_ population derived from the *acl83* × ZH11 cross. A total of 895 F_2_ individuals exhibiting an abaxially curled leaf phenotype were selected for linkage mapping analysis. On the other hand, leaf blades from 30 F_2_ individuals with the mutant phenotype in the *acl83* × 676R population were used to prepare DNA pools, which were subjected to bulk sequencing (Novogene Co., Ltd., Beijing, China). MutMap analysis was then performed as previously described (Abe et al., 2012), using the wild‐type 676R genome as the reference.

### Vector construction and plant transformation

For complementation and overexpression assay, the 1374‐bp full‐length coding sequence of *ACL83* (*LOC_Os05g32110*) was amplified from cDNA of the wild‐type 676R and inserted into the binary vectors pCAMBIA2300‐Actin1 and pCAMBIA2300‐35S, respectively. The complementation construct was transformed into the near‐isogenic line of *acl83* in the ZH11 genetic background (NIL^ZH11^), while the overexpression construct was introduced into wild‐type ZH11 using the *Agrobacterium*‐mediated method. For genome editing, CRISPR/Cas9 vectors were constructed as described previously (Cong et al., 2013). The targeting sequences for *ACL83* (SG1 and SG2), ROC5 (SG3 and SG4), and both genes (SG1–SG4) were synthesized and annealed to form the oligo adapters. These constructs were introduced into the ZH11 genetic background via *Agrobacterium*‐mediated transformation as described previously (Hiei et al., 1994). *ACL83* and ROC5 were individually knocked out to generate single mutants. Meanwhile, *ACL83* and *ROC5* were simultaneously knocked out to create an *acl83*/*roc5* double mutant.

### RNA isolation and qRT‐PCR analysis

Total RNA was extracted using Total RNA Extraction Reagent (R401‐01, Vazyme, China) from rice leaf blades, roots, leaf sheaths, stems, and young panicles collected at various developmental stages. First‐strand cDNA was synthesized from 2 μg total RNA with HiScript II Q RT SuperMix (R223‐01, Vazyme, China). qRT‐PCR was performed using SYBRPrime (BG0014, Bioground, China) on a CFX96 real‐time PCR system (qTOWER3 G, Jena, Germany). The PCR reaction mixture and program were as described previously (Guo et al., 2025). The *Actin1* gene was used as the internal control. Relative expression levels were calculated using the 2^−ΔΔCT^ method from at least three independent biological replicates.

### Subcellular localization

The 1371‐bp full‐length coding sequence of *ACL83* was amplified from the wild‐type cDNA of 676R, and inserted into the pCAMBIA2300‐GFP vector to generate the *ACL83*‐GFP fusion construct. The *ACL83*‐GFP construct and the empty vector (control) were each co‐transformed with the plasma membrane marker (RAC3‐mCherry) (Chen et al., 2010) into rice protoplasts. The transformation procedure was performed as described previously (Zhang et al., 2011). After overnight incubation in the dark, fluorescent signals were observed using a laser scanning confocal microscope (STELLARIS STED, Leica, Germany).

### Yeast one‐hybrid assay

As described previously (Liang et al., 2014), the full‐length coding sequence (CDS) of *ROC5* was amplified from *japonica* rice variety ZH11 using the primers containing homologous arms and an *EcoR*I restriction site, and then inserted into the pJG4‐5 vector to generate AD‐ROC5. The *ACL83p*:*LacZ* reporter was constructed by PCR amplification of the *ACL83* promoter (2071 bp upstream of ATG) using primers containing homologous arms and an *Xho*I restriction site, followed by cloning into the pLacZi2*μ* vector. The *ACL83p*:*LacZ* reporter plasmid was co‐transformed with the AD‐ROC5 into the yeast strain EGY48. Transformants were selected on SD/‐Trp/‐Ura medium and incubated at 28°C for 2–4 days. Single colonies were streaked onto SD/‐Trp/‐Ura plates supplemented with X‐gal and incubated at 28°C in the dark for 1–2 days. Similar results were obtained in three independent biological replicates; a representative result is shown.

### Dual‐LUC assay

Effector constructs were generated by cloning the full‐length coding sequence of *ROC5* into pCAMBIA2300 under the control of the CaMV 35S promoter. For the reporter, a 2071‐bp fragment of the *ACL83* promoter was inserted into pGreenII 0800‐LUC double reporter vector to generate the *ACL83p*::LUC construct. In this system, *Renilla* luciferase (REN), driven by the CaMV 35S promoter, served as an internal control. Effector and reporter plasmids were co‐delivered into *Nicotiana benthamiana* leaf epidermal cells via *Agrobacterium*‐mediated infiltration or co‐introduced into rice protoplasts by PEG‐mediated transformation, as described previously (Tao et al., 2021). For *N. benthamiana* assay, 1 mM D‐luciferin potassium salt (40902ES01, Yeasen, China) was smeared onto the leaf surface, and luminescence was captured using a FUSION‐FX7 imaging system (VILBER LOURMAT, Frence). Firefly luciferase (LUC) and REN activities were quantified with the Dual Luciferase Reporter Assay Kit (DL101‐01, Vazyme, China) on a GloMax® 96 Microplate Luminometer (Promega, America) according to the manufacturer's instruction. At least three independent replicates were performed.

### DAP‐qPCR assay

A prokaryotic expression system was employed to produce recombinant ROC5 protein for DNA affinity purification (DAP) assay. The full‐length coding sequence of *ROC5* was amplified from the ZH11 and inserted into the pCold‐TF vector to generate a TF‐His‐ROC5 fusion construct. The fusion protein was expressed in *Escherichia coli* BL21 and purified by Ni^2+^‐affinity chromatography using Ni‐TED 6FF resin (Sangon, China). The corresponding His‐TF protein (lacking ROC5) was prepared and purified in parallel as a negative control. Genomic DNA was extracted from 3‐week‐old seedlings of ZH11 and fragmented to approximately 100–500 bp by ultrasonication. To interrogate ROC5 binding across the *ACL83* promoter, 12 primer pairs generating 100–300 bp amplicons were designed to tile the promoter region. The DAP procedure was performed as described previously (Li, Zhu, et al., 2017; Tao et al., 2021). DNA eluted from the DAP assay was quantified by qPCR using SYBRPrime (BG0014, Bioground, China) on a CFX96 real‐time PCR system (qTOWER3 G, Jena, Germany), with cycling conditions as described previously (Li, Zhu, et al., 2017). The DNA sequence of the *UBQ5* promoter was used as an internal control. For enrichment analysis, input DNA fragments (without DAP incubation) were used as a negative control and normalized to 1.

### EMSA

Electrophoretic mobility shift assay (EMSA) was performed as described previously (Sun et al., 2021). Purified TF‐His‐ROC5 protein was used for the binding reactions. A biotin‐labelled 59‐bp DNA probe encompassing the L1‐box *cis*‐element from the *ACL83* promoter was synthesized by Tsingke (Beijing, China) and used in the assay. The corresponding unlabeled probe was used as a competitor probe, and purified TF‐His alone protein was used as a negative control. EMSA reactions and chemiluminescent detection were carried out using the Chemiluminescent EMSA Kit (GS009, Beyotime, China) according to the manufacturer's instructions.

### Statistical analysis

Statistical analyses were performed as indicated in the figure legends.

#### ACCESSION NUMBERS

Sequence data from this article can be found in the MSU Rice Genome Annotation Project Database under the following accession numbers: *ACL83* (*LOC_Os05g32110*), *ROC5* (*LOC_Os02g45250*).

## AUTHOR CONTRIBUTIONS

PW and XD designed the research and supervised the study. CC performed most of the research and drafted the manuscript. RL conducted gene mapping and genetic transformation for functional complementation. JD, RL, HZ, DL, YX, JL, HS, YL, XY, JG, YZ, and BY performed the experiments and conducted fieldwork. CC, PW, XD, and CS analyzed data. XD, PW, and CC revised the manuscript. All authors reviewed the results and approved the final manuscript.

## CONFLICT OF INTEREST

The authors declare that they have no conflicts of interest.

## Supporting information


**Figure S1.** Sequence alignment and phylogenetic tree of ACL83 and its homologs.
**Figure S2.** Analysis of cell wall components in the leaves of *acl83*.
**Figure S3.** Construction of the *acl83* near‐isogenic line (NIL^ZH11^) in the ZH11 genetic background.
**Figure S4.** Identification of transgenic plants (TP) for genetic complementation and comparison of agronomic traits among ZH11, NIL^ZH11^, and TP.
**Figure S5.** Sequencing chromatograms and protein sequence alignment between ZH11 and *ACL83* knockout lines.
**Figure S6.** Comparison of agronomic traits between ZH11 and *ACL83*‐knockout lines.
**Figure S7.** Phenotypic analysis of *ACL83*‐overexpression lines.
**Figure S8.** Activation of the *ACL83* promoter by ROC5 in protoplasts.
**Figure S9.** Expression and purification of *ROC5* recombinant protein in *Escherichia coli* for DAP‐qPCR and EMSA.
**Figure S10.** Sequencing chromatograms and protein sequence alignment between ZH11 and *ROC5*‐knockout lines.
**Figure S11.** Sequencing chromatograms and protein sequence alignment of *ACL83* and *ROC5* between ZH11 and *acl83*/*roc5* double‐mutant lines.
**Figure S12.** Comparison of agronomic traits among ZH11, *roc5* ^ZH11^, *acl83* ^ZH11^, and *acl83*/*roc5* double mutant.

**Table S1.** Genetic segregation of the abaxially curled leaf trait in an F_2_ population derived from the cross between the *acl83* mutant and the wild‐type 676R.
**Table S2.** Putative genes within the 196‐kb mapping region.
**Table S3.** Primers used in the study.

## Data Availability

All relevant data can be found within the manuscript and its Supplementary Information materials.
