# Supplementary material for: A COBRA‐like protein ACL83 acts downstream of transcription factor ROC5 to control abaxial leaf curling in rice
Source: Plant J. 2026 Sep 28;127(6):e71144. doi: 10.1111/tpj.71144 (PMC13618896; doi:10.1111/tpj.71144)
Supplement: Supplementary file 1 [file TPJ-127-0-s001.pdf]

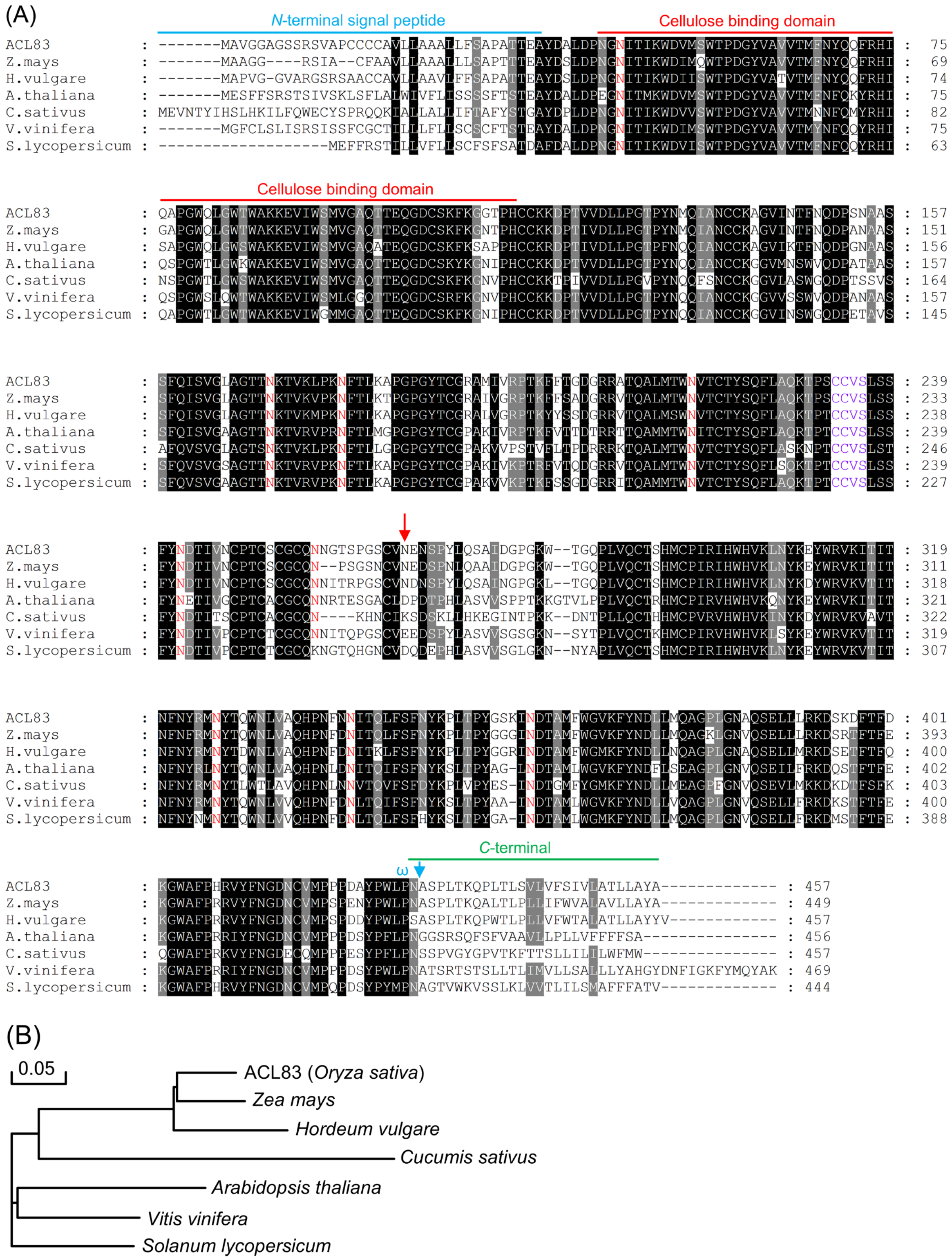

Figure S1. Sequence alignment and phylogenetic tree of ACL83 and its homologs.

(A) Multiple sequence alignment of ACL83 and its homologs. Sequences were aligned using MEGA9 and further optimized by GeneDoc. Blue overline indicates the predicted *N-terminal* signal peptides; green overline indicates the *C-terminal* region, including the predicted ω-site and hydrophobic tail; red overlines indicate the predicted cellulose-binding domain. Purple uppercase letters indicate the conserved CCVS motif, and red uppercase letters indicate putative N-glycosylation sites. The red arrow indicates the mutation site in the *acl83* mutant.

(B) Phylogenetic analysis of ACL83 and its homologs. The unrooted neighbor-joining tree using percentage

identities was constructed based on a multiple sequence alignment produced with the program DNAMAN. Accession numbers for the respective protein sequences are as follows: *Oryza sativa* (ACL83, LOC_Os05g32110), *Zea mays* (NP_001105969.1), *Hordeum vulgare* (KAI4984924.1), *Cucumis sativus* (XP_011648659.1), *Arabidopsis thaliana* (AT5G60920), *Vitis vinifera* (CAN68249.1), and *Solanum lycopersicum* (NP_001265883.1). Scale represents percentage substitution per site.

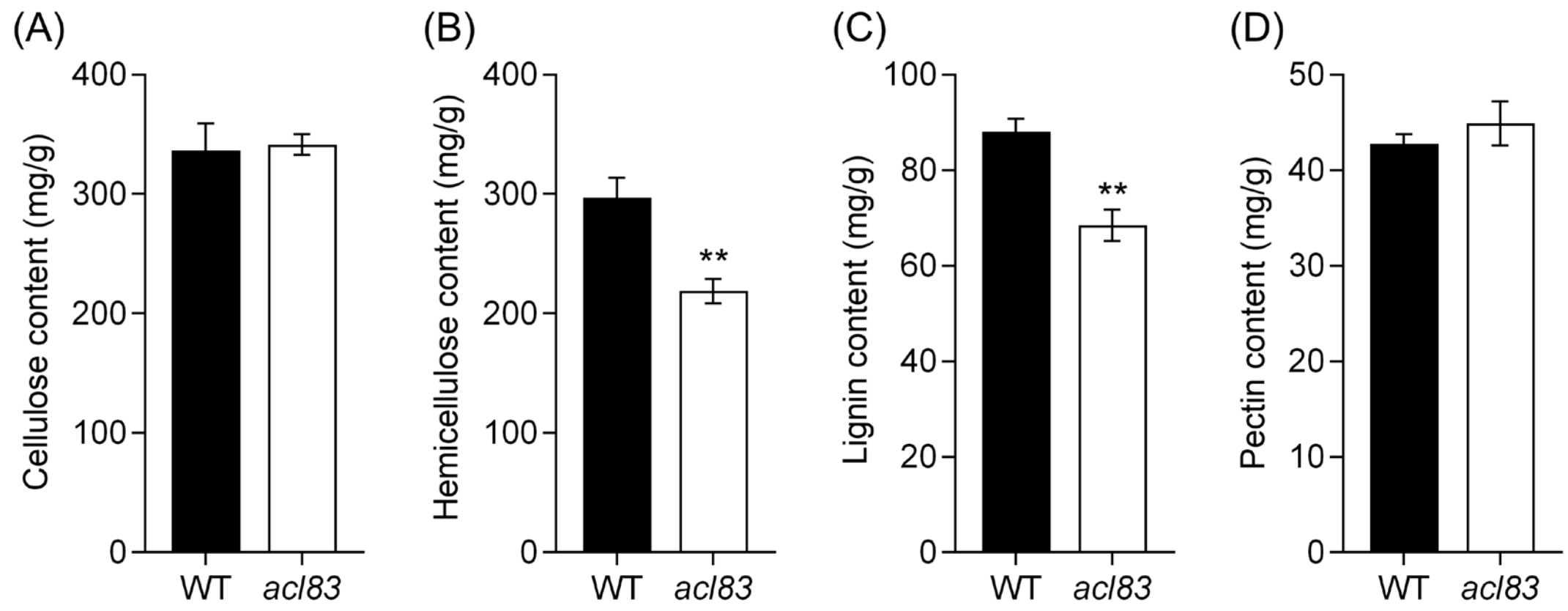

Figure S2. Analysis of cell wall components in the flag leaves of *acl83*.

(A–D) Contents of cellulose (A), hemicellulose (B), lignin (C), and pectin (D) in leaves of the wild type (WT) and the *acl83* mutant at the heading stage. Values are means ± SD of three biological replicates. Asterisks indicate significant differences between *acl83* and WT by Student's t-test ($^{**}P < 0.01$).

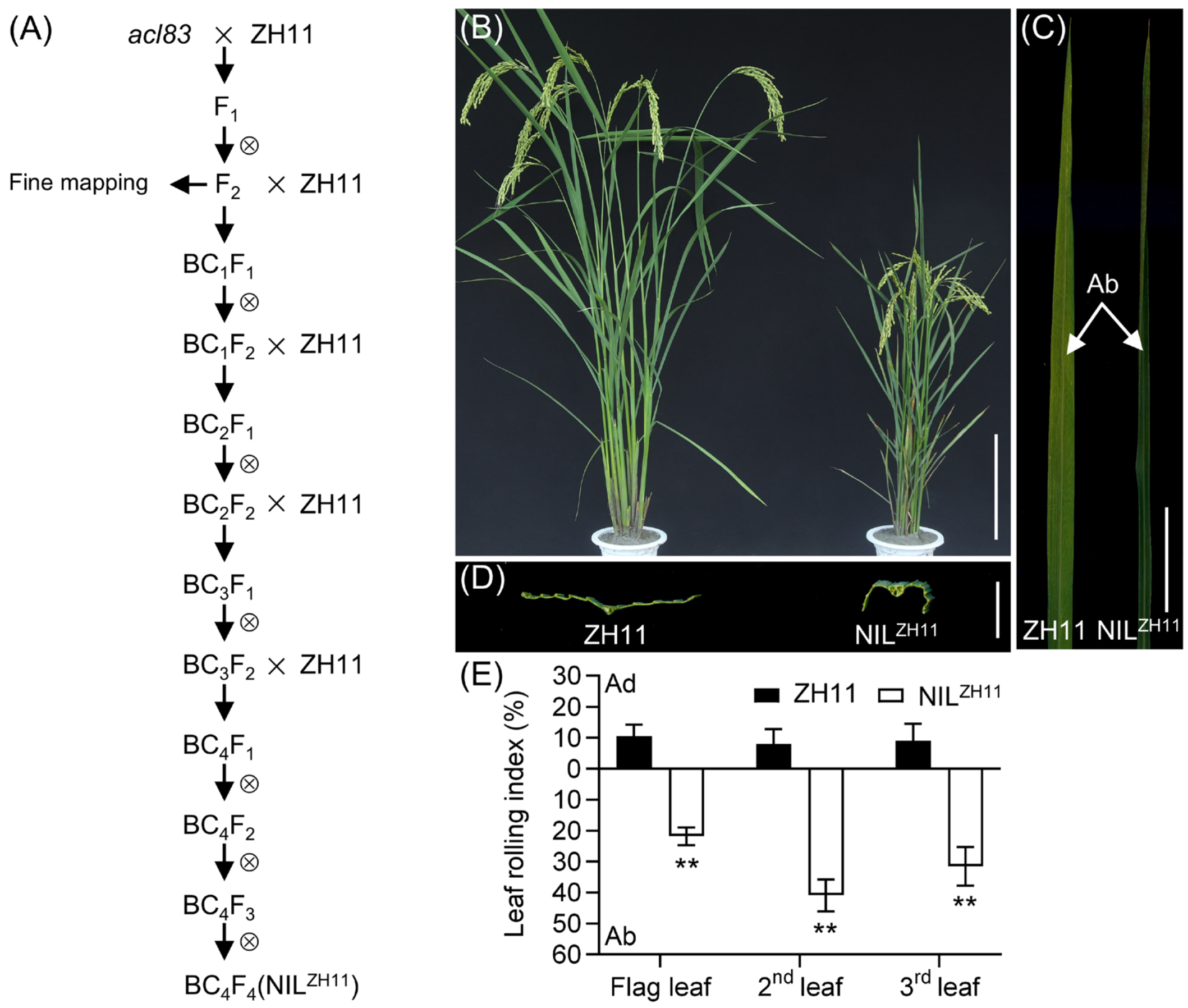

Figure S3. Construction of the *acl83* near-isogenic line (NIL^{ZH11}) in the ZH11 genetic background.

(A) Schematic diagram of the breeding scheme used to construct the *acl83* near-isogenic line (NIL^{ZH11}). Individual plants exhibiting the *acl83* leaf-rolling phenotype and the plant architecture similar to *japonica* cultivar ZH11 were selected from the F_2, BC_1F_2, BC_2F_2, and BC_3F_2 populations and backcrossed to ZH11. The symbols × and ⊗ indicate crossing (cross-pollination) and selfing (self-pollination), respectively.

(B) Plant architectures of ZH11 and NIL^{ZH11} at the grain-filling stage. Scale bar, 20 cm.

(C) Leaf phenotype of the top 2nd leaves in ZH11 and NIL^{ZH11} at the grain-filling stage. Ab, abaxial. Scale bar, 5 cm.

(D) Transverse sections of the top 2nd leaves in ZH11 and NIL^{ZH11} at the grain-filling stage. Scale bar, 0.5 cm.

(E) Leaf rolling indices of the flag leaf, second leaf and third leaf from the top in ZH11 and NIL^{ZH11} at the grain-filling stage. Ad and Ab indicate adaxial and abaxial rolling respectively. Values are means ± SD of three biological replicates. Asterisks indicate significant differences between ZH11 and NIL^{ZH11} by Student's *t*-test ($^{**}P < 0.01$).

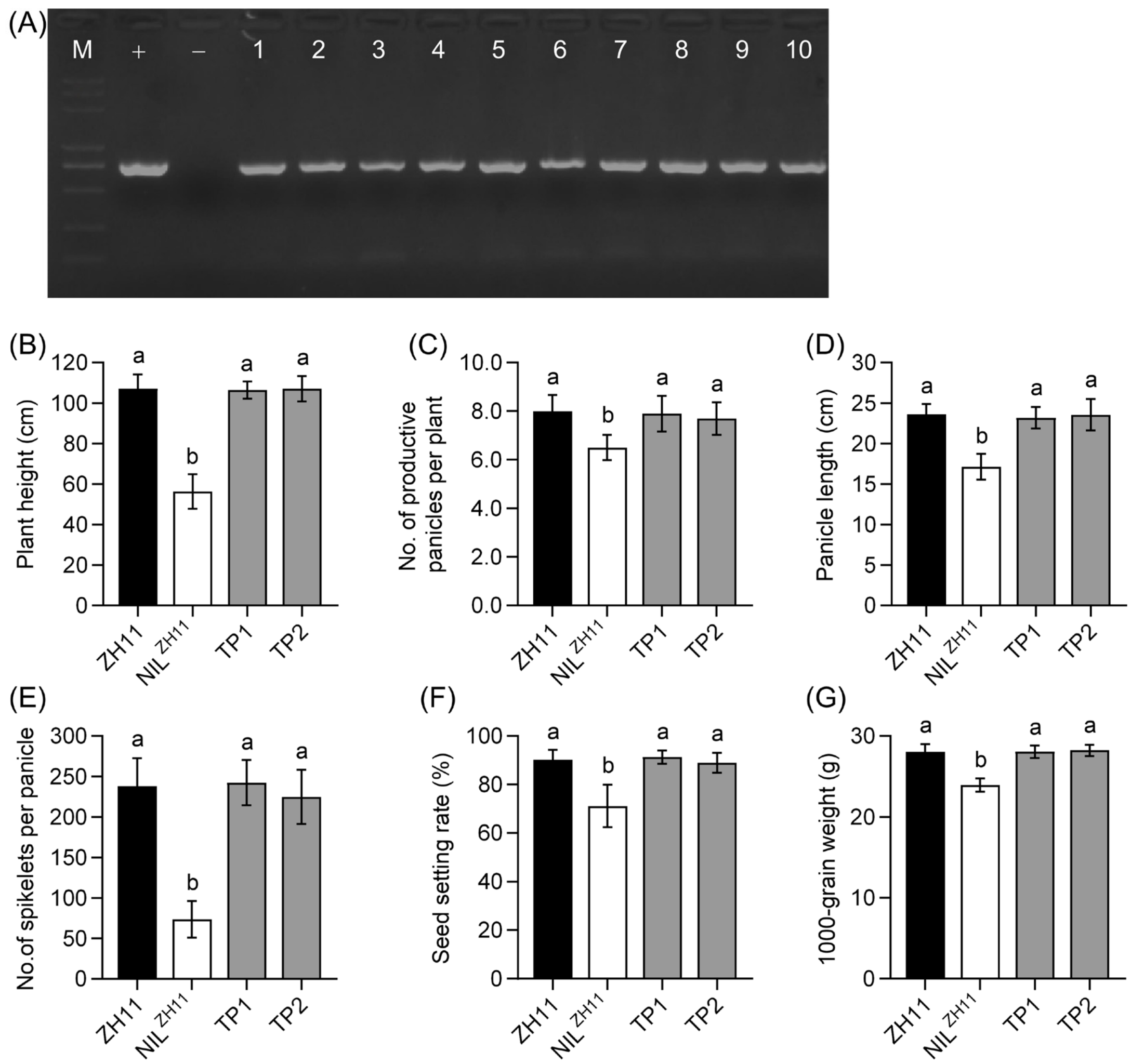

Figure S4. Identification of transgenic plants (TP) for genetic complementation and comparison of agronomic traits among ZH11, NIL^{ZH11} and TP.

(A) Identification of transgenic lines by PCR. M, 5000 bp-III DNA Marker; +, pCAMBIA2300-*ACL83* plasmid (positive control); -, NIL^{ZH11} (negative control); 1-10, positive transgenic plants (TP).

(B–G) Comparison of plant height, number of productive panicles per plant, panicle length, number of spikelets per panicle, seed setting rate, and 1000-grain weight at the maturity stage. Values are means ± SD of three biological replicates. Statistical significance was determined by one-way ANOVA with Tukey's multiple-comparison test ($P < 0.05$), and different lowercase letters indicate significant differences.

(A)

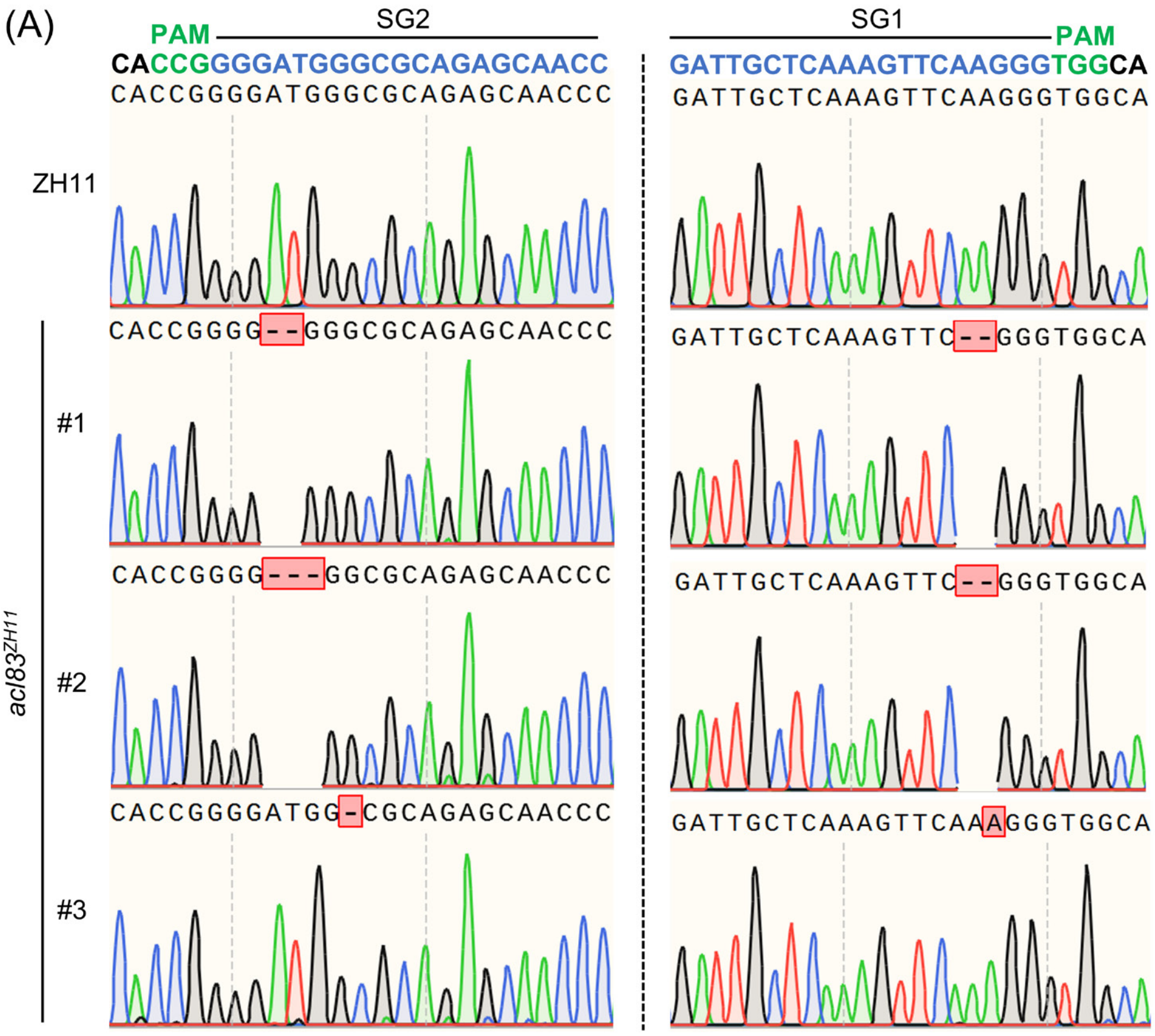

(B)

```
ZH11 : MAVGGAGSSRSVAPCCCCAVLLAAALLFSAPATTEAYDALDPNGNITIKWDVMSWTPDGYVAVVTMFNYQQFRHIQAPGWQLGWTWAKKEVIWSMVGAQT : 100
#1   : MAVGGAGSSRSVAPCCCCAVLLAAALLFSAPATTEAYDALDPNGNITIKWDVMSWTPDGYVAVVTMFNYQQFRHIQAPGWQLGWTWAKKEVIWSMVGAQT : 100
#2   : MAVGGAGSSRSVAPCCCCAVLLAAALLFSAPATTEAYDALDPNGNITIKWDVMSWTPDGYVAVVTMFNYQQFRHIQAPGWQLGWTWAKKEVIWSMVGAQT : 100
#3   : MAVGGAGSSRSVAPCCCCAVLLAAALLFSAPATTEAYDALDPNGNITIKWDVMSWTPDGYVAVVTMFNYQQFRHIQAPGWQLGWTWAKKEVIWSMVGAQT : 100

ZH11 : TEQGDCSKFKGGTPHCCKKDPTVVDLLPGTPYNMQIANCCKAGVINTFNQDPSNAASSFQISVGLAGTTNKTVKLPKNFTLKAPGPGYTCGRAMIVRPTK : 200
#1   : TEQGDCSKFGWHAPLLQEGSHSC*---------------------------------------------------------------------------- : 123
#2   : TEQGDCSKFGWHAPLLQEGSHSC*---------------------------------------------------------------------------- : 123
#3   : TEQGDCSKFKGWHAPLLQEGSHSC*--------------------------------------------------------------------------- : 124

ZH11 : FFTGDGRRATQALMTWNVTCTYSQFLAQKTPSCCVSLSSFYNDTIVNCPTCSCGCQNNGTSPGSCVNENSPYLQSAIDGPGKWTGQPLVQCTSHMCPIRI : 300
#1   : ---------------------------------------------------------------------------------------------------- :   -
#2   : ---------------------------------------------------------------------------------------------------- :   -
#3   : ---------------------------------------------------------------------------------------------------- :   -

ZH11 : HWHVKLNYKEYWRVKITITNFNYRMNYTQWNLVAQHPNFNNITQLFSFNYKPLTPYGSKINDTAMFWGVKFYNDLLMQAGPLGNAQSELLLRKDSKDFTF : 400
#1   : ---------------------------------------------------------------------------------------------------- :   -
#2   : ---------------------------------------------------------------------------------------------------- :   -
#3   : ---------------------------------------------------------------------------------------------------- :   -

ZH11 : DKGWAFPHRVYFNGDNCVMPPPDAYPWLPNASPLTKQPLTLSVLVFSIVLATLLAYA* : 457
#1   : --------------------------------------------------------- :   -
#2   : --------------------------------------------------------- :   -
#3   : --------------------------------------------------------- :   -
```

Figure S5. Sequencing chromatograms and protein sequence alignment between ZH11 and *ACL83* knockout lines.
(A) Sanger sequencing chromatograms of the CRISPR/Cas9 target sites SG1 and SG2 in ZH11 and three knockout lines (*acl83*^{ZH11} #1, #2 and #3). Blue letters indicate target sequences, and green letters indicate PAM sites.
(B) Alignment of ACL83 protein sequences between ZH11 and *ACL83*-knockout lines. Black boxes indicate identical amino acid residues, and red asterisks indicate predicted premature stop codons.

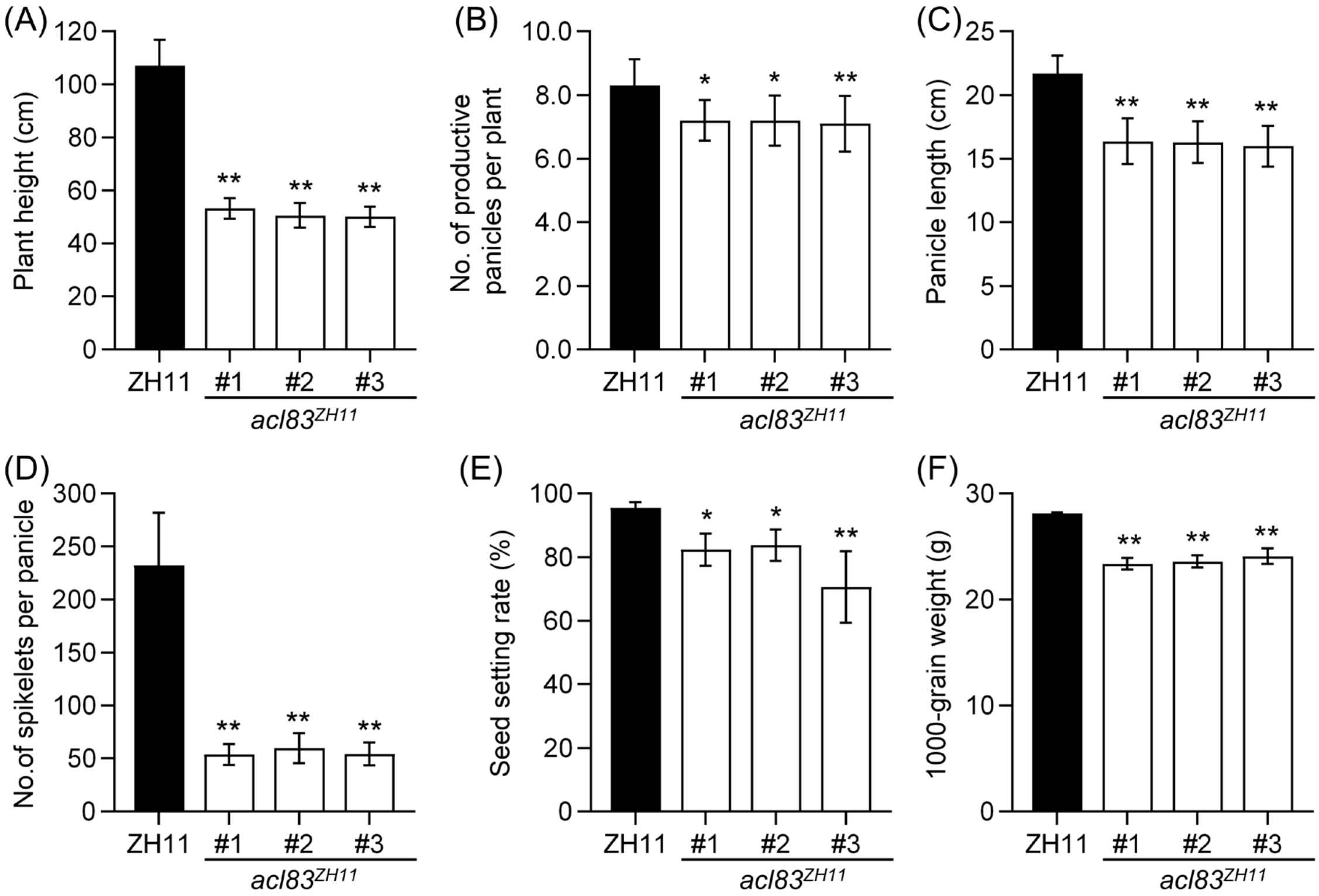

Figure S6. Comparison of agronomic traits between ZH11 and *ACL83*-knockout lines.

(A–F) Plant height, number of productive panicles per plant, panicle length, number of spikelets per panicle, seed setting rate, and 1000-grain weight at the maturity stage. Values are means ± SD of three biological replicates. Asterisks indicate significant differences from ZH11 as determined by Student's *t*-test ($^{*}P < 0.05$, $^{**}P < 0.01$).

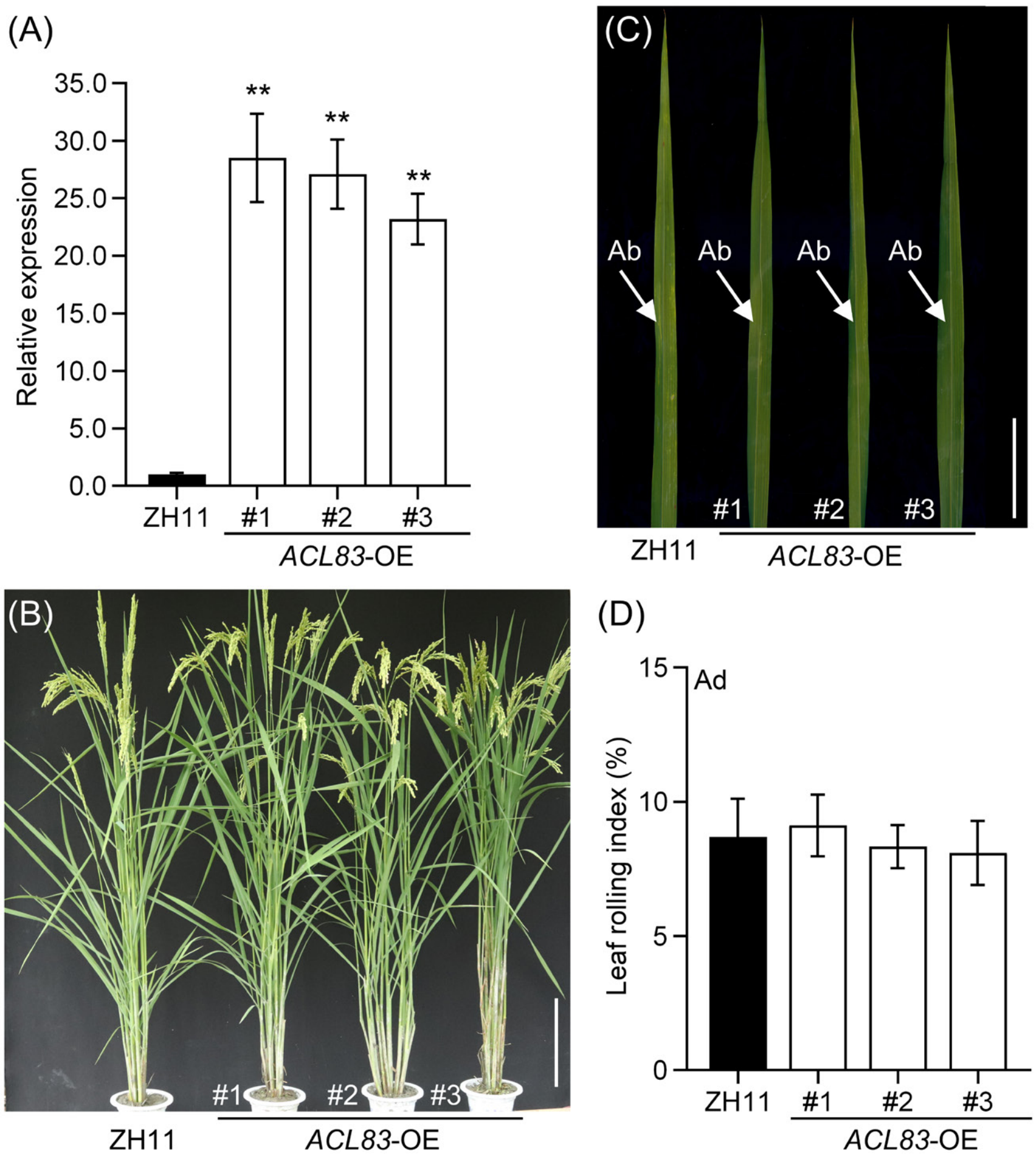

Figure S7. Phenotypic analysis of *ACL83*-overexpression lines.

(A) Relative expression levels of *ACL83* in ZH11 and three independent *ACL83*-overexpression (*ACL83*-OE) lines (#1, #2, and #3) at the seedling stage. *ACL83* expression in ZH11 was normalized to 1.0, and the relative expression levels in *ACL83*-OE lines were determined accordingly.

(B) Plant architecture of ZH11 and *ACL83*-OE lines at the grain-filling stage. Scale bar, 20 cm.

(C) Leaf phenotype of the top 2nd leaves in ZH11 and *ACL83*-OE lines at the grain-filling stage. Ab, abaxial. Scale bar, 5 cm.

(D) Leaf rolling indices of the top 2nd leaves in ZH11 and *ACL83*-OE lines at the grain-filling stage. Ad, Adaxial. Values are means ± SD of three biological replicates. In (a), asterisks indicate significant differences between ZH11 and *ACL83*-OE lines by Student's *t*-test ($^{**}P < 0.01$).

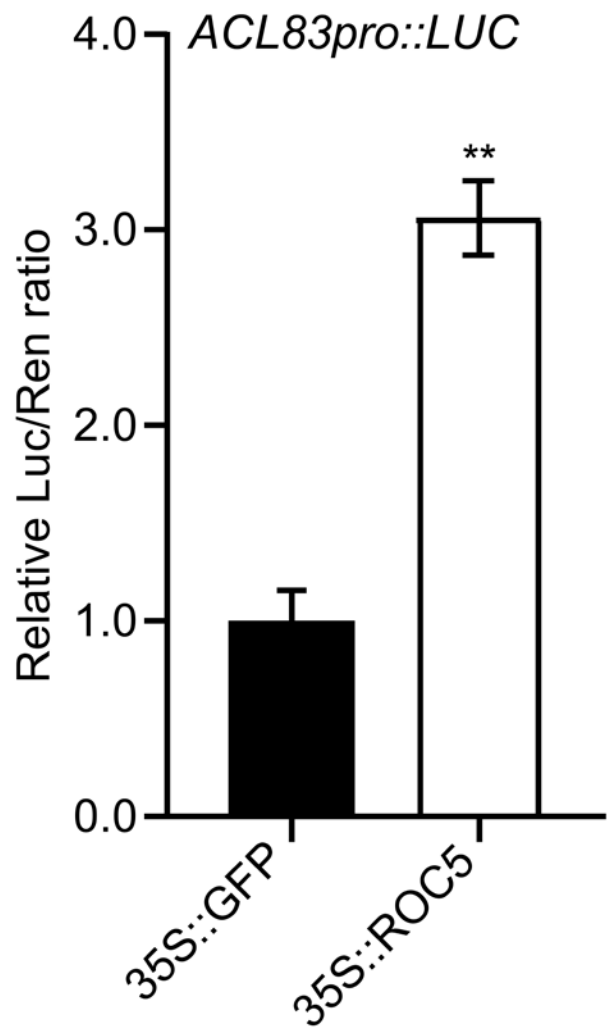

Figure S8. Activation of the *ACL83* promoter by *ROC5* in protoplasts.

Effectors pCAMBIA2300-GFP-*ROC5* and pCAMBIA2300-GFP (control) together with the reporter construct *ACL83pro*::LUC-pGreenII-0800 were co-transformed into rice protoplasts. The LUC/REN ratio of *ACL83pro*::LUC-pGreenII-0800 co-transformed with pCAMBIA2300-GFP was set to 1.0. Values are means ± SD of four biological replicates. Asterisks indicate significant differences from GFP control as determined by Student's *t*-test ($^{**}P < 0.01$).

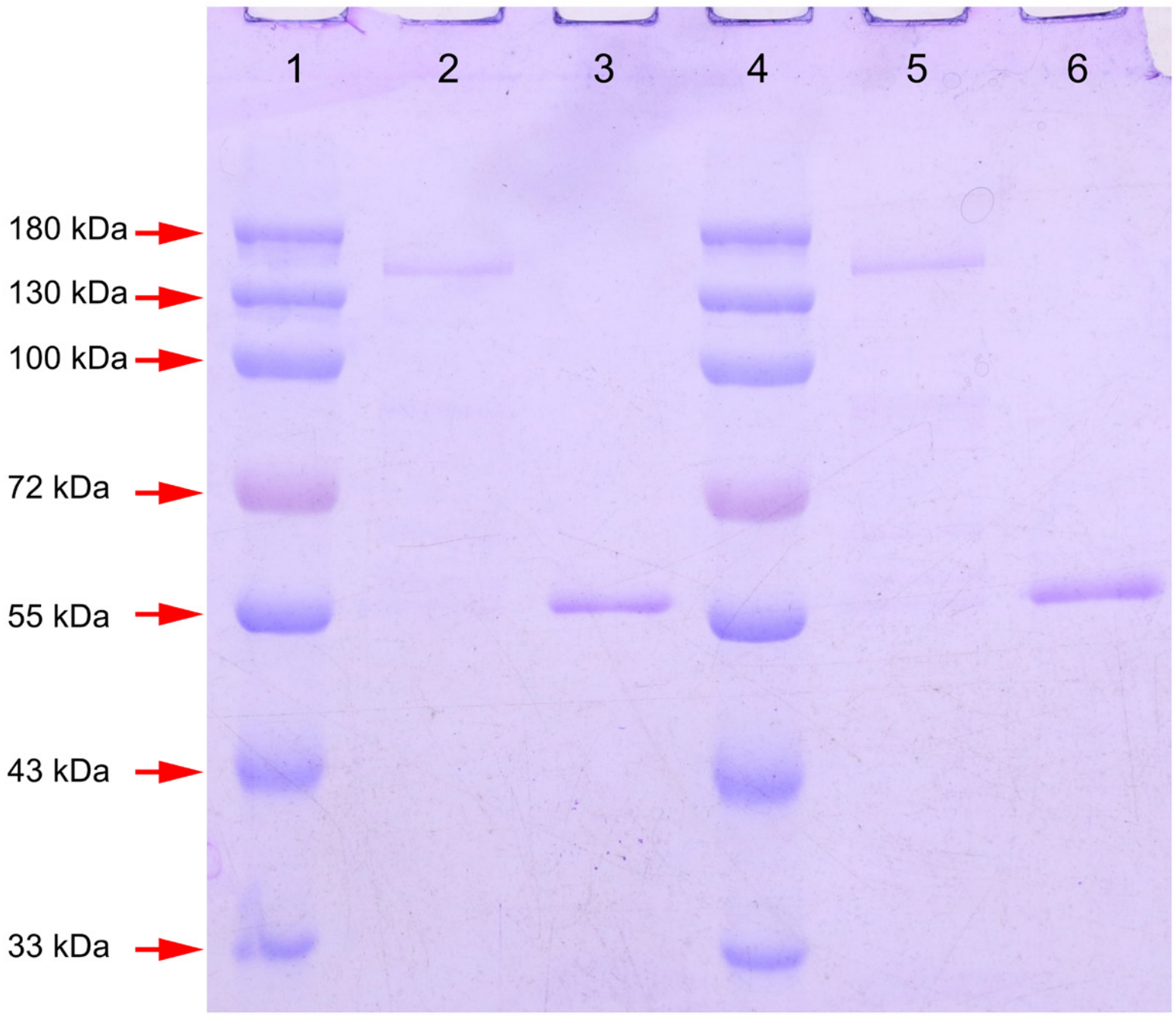

Figure S9. Expression and purification of ROC5 recombinant protein in *Escherichia coli* for DAP-qPCR and EMSA.

SDS-PAGE electrophoresis analysis (10% polyacrylamide gel) of the His-pCold-TF-ROC5 fusion protein constructed with the His-pCold TF vector and expressed in *Escherichia coli*. Lanes 1 and 4 indicate protein Marker, Lanes 2 and 5 indicate purified His-pCold-TF-ROC5, and lanes 3 and 6 indicate the empty pCold TF vector. TF denotes the trigger factor solubility tag. The predicted molecular weights of His-pCold-TF-ROC5 and His-pCold TF are 139.2 and 54.4 kDa, respectively.

(A)

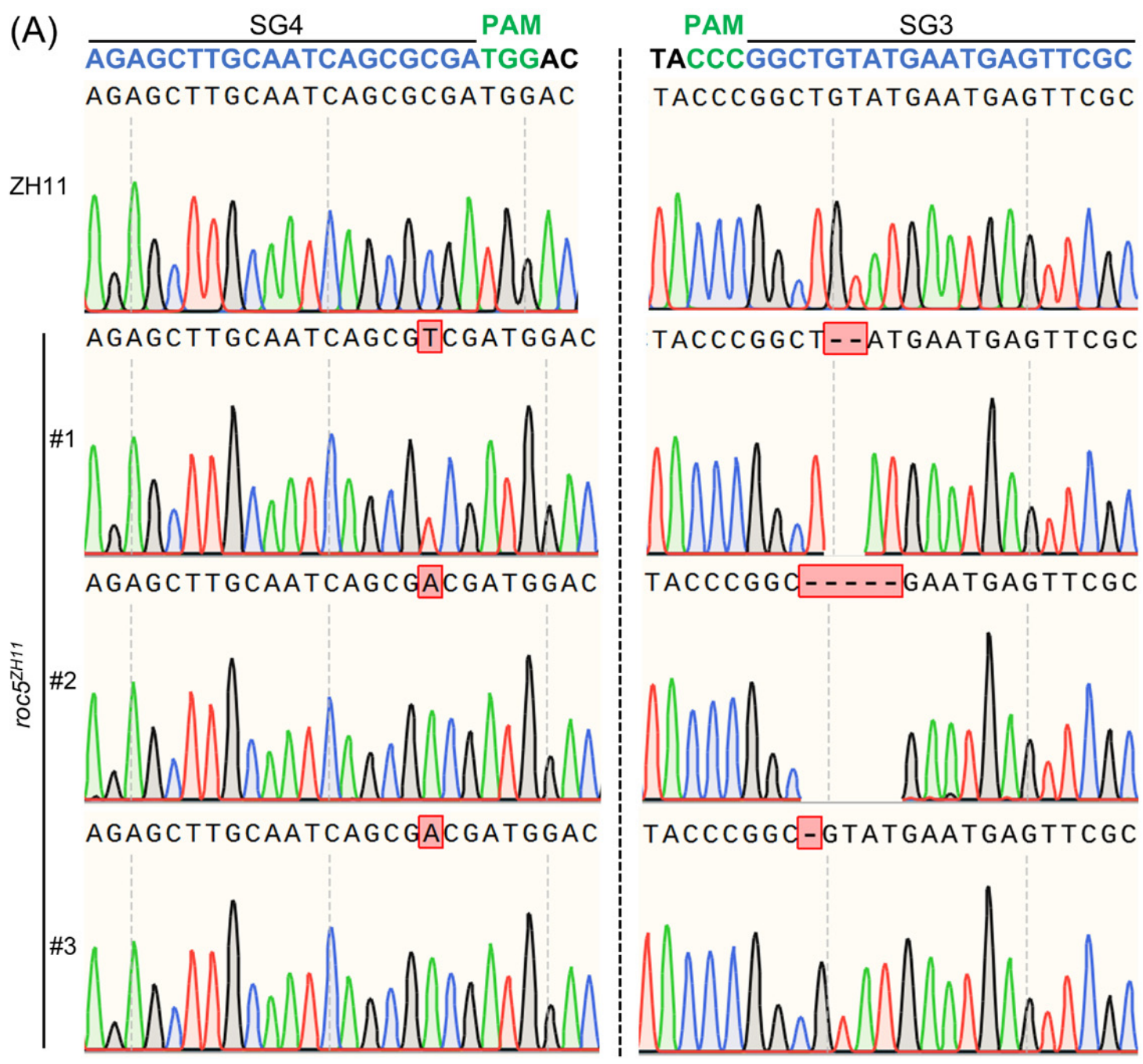

(B)

```
ZH11 : MSFGGLFDGGGGGGMQFPFASGFASSPALSLALDNAGGGIGGRMLGGGAGAGSSAGGAMTRDTEAENDSRSGSDHLDAISAAGEDDVEDAEPSNSRKRKK : 100
#1   : MSFGGLFDGGGGGGMQFPFASGFASSPALSLALDNAGGGIGGRMLGGGAGAGSSAGGAMTRDTEAENDSRSGSDHLDAISAAGEDDVEDAEPSNSRKRKK : 100
#2   : MSFGGLFDGGGGGGMQFPFASGFASSPALSLALDNAGGGIGGRMLGGGAGAGSSAGGAMTRDTEAENDSRSGSDHLDAISAAGEDDVEDAEPSNSRKRKK : 100
#3   : MSFGGLFDGGGGGGMQFPFASGFASSPALSLALDNAGGGIGGRMLGGGAGAGSSAGGAMTRDTEAENDSRSGSDHLDAISAAGEDDVEDAEPSNSRKRKK : 100

ZH11 : RYHRHTPQQIQELEALFKECPHPDEKQRAELSRRLSLDARQVKFWFQNRRTQMKTQLERHENALLKQENDKLRAENMTIREAMRSPMCGSCGSPAMLGEV : 200
#1   : RYHRHTPQQIQELEALFKECPHPDEKQRAELSRRLSLDARQVKFWFQNRRTQMKTQLERHENALLKQENDKLRAENMTIREAMRSPMCGSCGSPAMLGEV : 200
#2   : RYHRHTPQQIQELEALFKECPHPDEKQRAELSRRLSLDARQVKFWFQNRRTQMKTQLERHENALLKQENDKLRAENMTIREAMRSPMCGSCGSPAMLGEV : 200
#3   : RYHRHTPQQIQELEALFKECPHPDEKQRAELSRRLSLDARQVKFWFQNRRTQMKTQLERHENALLKQENDKLRAENMTIREAMRSPMCGSCGSPAMLGEV : 200

ZH11 : SLEEQHLRIENARLKDELNRVCALATKFLGKPISLLSPPPLLQPHLSLPMPNSSLELAIGGIGGLGSLGTLPGCMNEFAGGVSSPMGTVITPARATGAAI : 300
#1   : SLEEQHLRIENARLKDELNRVCALATKFLGKPISLLSPPPLLQPHLSLPMPNSSLELAIGGIGGLGSLGTLPGYE*---------------------- : 275
#2   : SLEEQHLRIENARLKDELNRVCALATKFLGKPISLLSPPPLLQPHLSLPMPNSSLELAIGGIGGLGSLGTLPGE*----------------------- : 274
#3   : SLEEQHLRIENARLKDELNRVCALATKFLGKPISLLSPPPLLQPHLSLPMPNSSLELAIGGIGGLGSLGTLPGV*----------------------- : 274

ZH11 : PSLVGNIDRSVFLELAISAMDELVKMAQMDDPLWVPALPGSPSKEVLNFEEYLHSFLPCIGMKPAGYVSEASRESGLVIIDNSLALVETLMDERRWSDMF : 400
#1   : ---------------------------------------------------------------------------------------------------- :   -
#2   : ---------------------------------------------------------------------------------------------------- :   -
#3   : ---------------------------------------------------------------------------------------------------- :   -

ZH11 : SCMIAKATVLEEVSTGIAGSRNGALLLMKAELQVLSPLVPIREVTFLRFCKQLAEGAWAVVDVSIDGLVRDHNSGTAPTGGNVKCRRVPSGCVMQDTPNG : 500
#1   : ---------------------------------------------------------------------------------------------------- :   -
#2   : ---------------------------------------------------------------------------------------------------- :   -
#3   : ---------------------------------------------------------------------------------------------------- :   -

ZH11 : YCKVTWVEHTEYDEASVHQLYRPLLRSGLAFGARRWLATLQRQCECLAILMSSATVTANDSTAISQEGKRSMLKLARRMTENFCAGVSASSAREWSKLDG : 600
#1   : ---------------------------------------------------------------------------------------------------- :   -
#2   : ---------------------------------------------------------------------------------------------------- :   -
#3   : ---------------------------------------------------------------------------------------------------- :   -

ZH11 : ATGSIGEDVRVMARKSVSEPGEPPGVVLSAATSVWVPVAPEKLFNFLRDEQLRAEWDILSNGGPMQEMTQIAKGQRDGNSVSLLRASAVSANQSSMLILQ : 700
#1   : ---------------------------------------------------------------------------------------------------- :   -
#2   : ---------------------------------------------------------------------------------------------------- :   -
#3   : ---------------------------------------------------------------------------------------------------- :   -

ZH11 : ETCTDASGSIVVYAPVDIPAMQLVMNGGDSTYVALLPSGFAILPDGPRIGATGYETGGSLLTVAFQILVNNQPTAKLTVESVETVNNLISCTIKKIKTAL : 800
#1   : ---------------------------------------------------------------------------------------------------- :   -
#2   : ---------------------------------------------------------------------------------------------------- :   -
#3   : ---------------------------------------------------------------------------------------------------- :   -

ZH11 : QCDA* : 804
#1   : ----- :   -
#2   : ----- :   -
#3   : ----- :   -
```

Figure S10. Sequencing chromatograms and protein sequence alignment between ZH11 and *ROC5*-knockout lines.

(A) Sanger sequencing chromatograms of the CRISPR/Cas9 target sites SG3 and SG4 in ZH11 and three knockout lines (*roc5*^{ZH11} #1, #2 and #3). Blue letters indicate target sequences, and green letters indicate PAM sites.

(B) Alignment of ROC5 protein sequences between ZH11 and *ROC5* knockout lines. Black boxes indicate identical amino acid residues, and red asterisks indicate predicted premature stop codons.

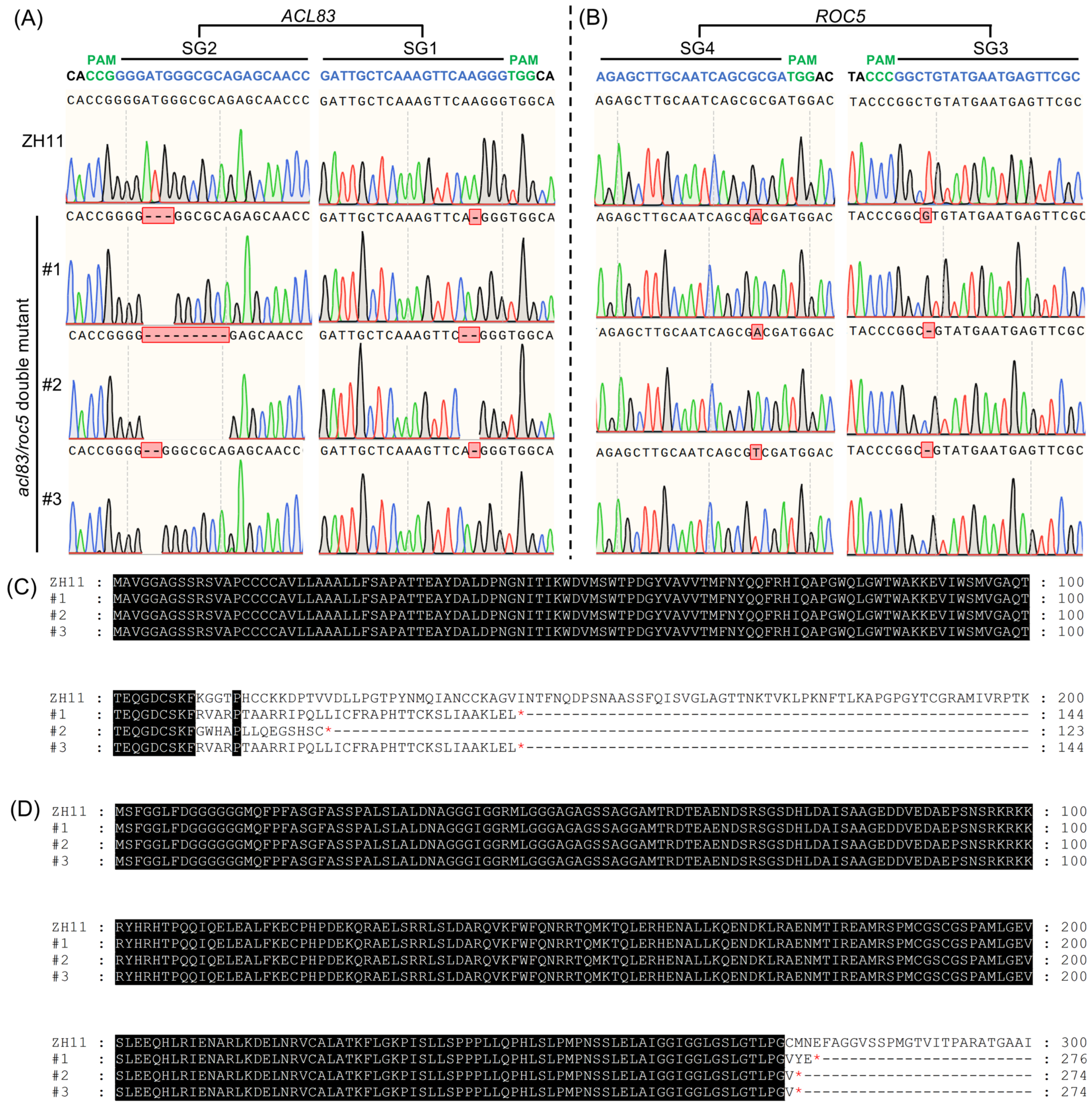

Figure S11. Sequencing chromatograms and protein sequence alignment of *ACL83* and *ROC5* between ZH11 and *acl83/roc5* double-mutant lines.

(A) Sanger sequencing chromatograms of the *ACL83* locus in ZH11 and three *acl83/roc5* double-mutant lines (#1, #2 and #3). Blue letters indicate target sequences, and green letters indicate PAM sites.

(B) Sanger sequencing chromatograms of the *ROC5* locus in ZH11 and three *acl83/roc5* double-mutant lines (#1, #2 and #3). Blue letters indicate target sequences, and green letters indicate PAM sites.

(C) Alignment of predicted *ACL83* protein sequences between ZH11 and the *acl83/roc5* double-mutant lines. Black boxes indicate identical amino acid residues, and red asterisks indicate predicted premature stop codons.

(D) Alignment of predicted *ROC5* protein sequences between ZH11 and the *acl83/roc5* double-mutant lines. Black boxes indicate identical amino acid residues, and red asterisks indicate predicted premature stop codons.

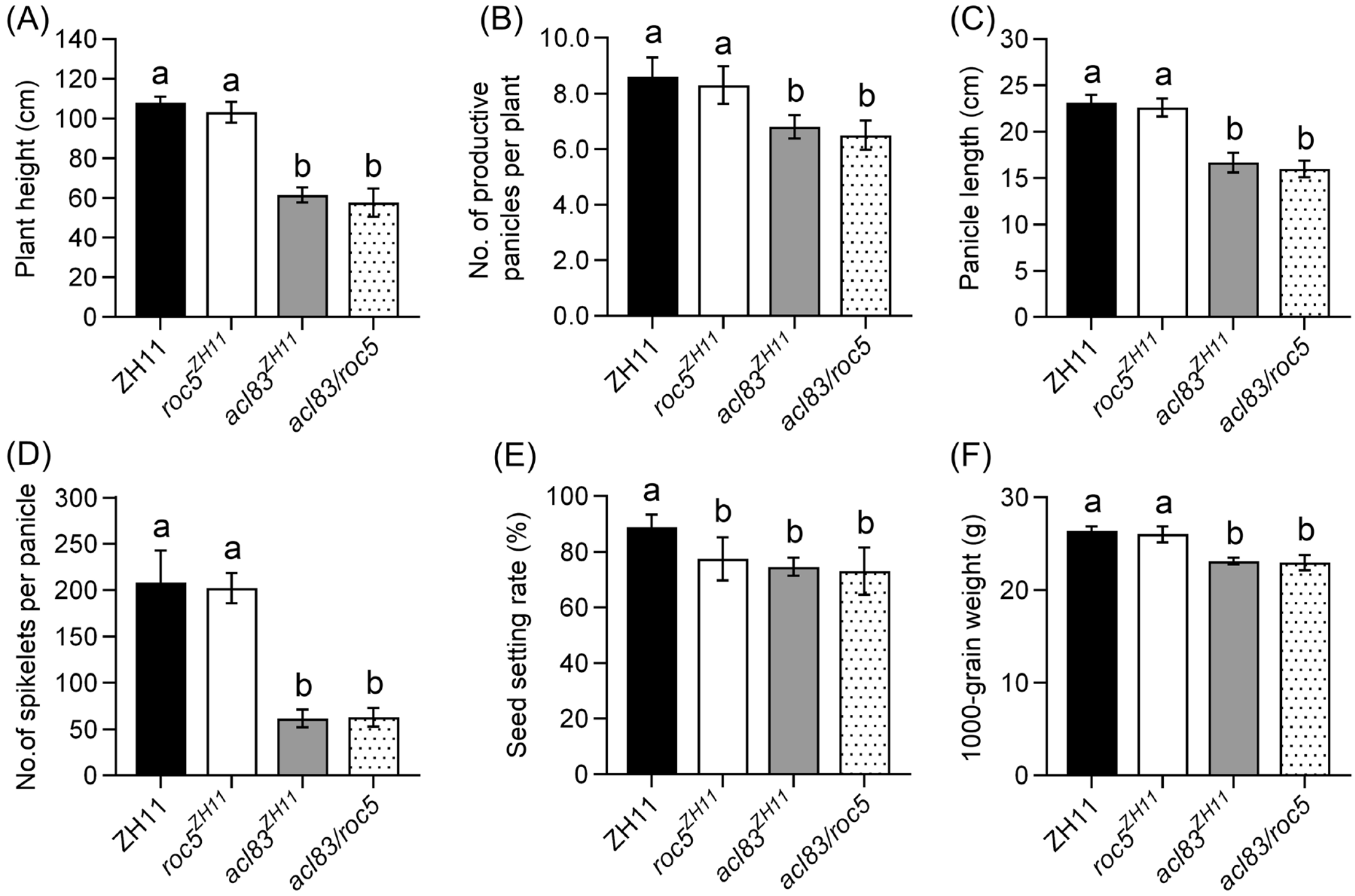

Figure S12. Comparison of agronomic traits among ZH11, *roc5*^{ZH11}, *acl83*^{ZH11} and *acl83/roc5* double mutant. (A–F) Plant height, number of productive panicles per plant, panicle length, number of spikelets per panicle, seed setting rate, and 1000-grain weight at the maturity stage. Values are means ± SD of three biological replicates. Statistical significance was determined by one-way ANOVA with Tukey's multiple-comparison test (P < 0.05), and different lowercase letters indicate significant differences.
