# Supplementary material for: A COBRA‐like protein ACL83 acts downstream of transcription factor ROC5 to control abaxial leaf curling in rice
Source: Plant J. 2026 Sep 28;127(6):e71144. doi: 10.1111/tpj.71144 (PMC13618896; doi:10.1111/tpj.71144)
Supplement: Supplementary file 2 [file TPJ-127-0-s002.docx]

**Table S1. Genetic segregation of the abaxially curled leaf trait in an F_2_ population derived from the cross between the *acl83* mutant and the wild-type 676R.**

| Combination | No. of total plants | No. of normal leaf plants | No. of abaxially curled leaf plants | Expected ratio | χ^2^ | χ^2^_0.05, (1)_ |
| --- | --- | --- | --- | --- | --- | --- |
| *acl83* × 676R | 672 | 522 | 150 | 3:1 | 2.43 | 3.84 |

**Table S2.** **Putative genes within the 196-kb mapping region.**

| Number | MSU Locus ID | Putative functions |
| --- | --- | --- |
| ORF1 | *LOC_Os05g31900* | expressed protein |
| ORF2 | *LOC_Os05g31910* | white-brown complex homolog protein 16, putative, expressed |
| ORF3 | *LOC_Os05g31920* | FYVE zinc finger domain containing protein, expressed |
| ORF4 | *LOC_Os05g31930* | retrotransposon protein, putative, unclassified, expressed |
| ORF5 | *LOC_Os05g31940* | retrotransposon protein, putative, unclassified, expressed |
| ORF6 | *LOC_Os05g31950* | retrotransposon protein, putative, Ty3-gypsy subclass, expressed |
| ORF7 | *LOC_Os05g31970* | expressed protein |
| ORF8 | *LOC_Os05g31990* | retrotransposon protein, putative, unclassified |
| ORF9 | *LOC_Os05g32000* | transposon protein, putative, unclassified |
| ORF10 | *LOC_Os05g32010* | hypothetical protein |
| ORF11 | *LOC_Os05g32020* | expressed protein |
| ORF12 | *LOC_Os05g32030* | transposon protein, putative, unclassified, expressed |
| ORF13 | *LOC_Os05g32040* | retrotransposon protein, putative, unclassified, expressed |
| ORF14 | *LOC_Os05g32050* | retrotransposon protein, putative, Ty3-gypsy subclass, expressed |
| ORF15 | *LOC_Os05g32060* | expressed protein |
| ORF16 | *LOC_Os05g32070* | LRP1, putative, expressed |
| ORF17 | *LOC_Os05g32080* | retrotransposon protein, putative, unclassified |
| ORF18 | *LOC_Os05g32090* | expressed protein |
| ORF19 | *LOC_Os05g32100* | expressed protein |
| **ORF20** | ***LOC_Os05g32110*** | **COBRA, putative, expressed** |
| ORF21 | *LOC_Os05g32120* | alpha 1,3-xylosyltransferase, putative, expressed |
| ORF22 | *LOC_Os05g32130* | expressed protein |
| ORF23 | *LOC_Os05g32140* | NAD dependent epimerase/dehydratase family protein, putative, expressed |
| ORF24 | *LOC_Os05g32150* | expressed protein |
| ORF25 | *LOC_Os05g32160* | expressed protein |
| ORF26 | *LOC_Os05g32170* | TKL_IRAK_CR4L.5 - The CR4L subfamily has homology with Crinkly4, expressed |
| ORF27 | *LOC_Os05g32180* | acetyltransferase, GNAT family, putative, expressed |

**Table S3.** **Primers used in the study.**

| Purpose | Primer name | Primer sequence (5'-3') | Remark |
| --- | --- | --- | --- |
| Gene mapping | RM163F | ATCCATGTGCGCCTTTATGAGGA | SSR |
|  | RM163R | CGCTACCTCCTTCACTTACTAGT | SSR |
|  | P1F | AATAGTTGCTGCATATTGCT | InDel |
|  | P1R | CTGCCACACATTGTCACCCT | InDel |
|  | P2F | ATAATTCAGTCAATACGCTT | InDel |
|  | P2R | TCAAAGGTGCAAGGAGCCAT | InDel |
|  | P3F | TGTACTTGAAGAACCACA | InDel |
|  | P3R | CACTGAACTGCTCATAAG | InDel |
|  | P4F | CGATACCGAAACTACTGA | InDel |
|  | P4R | CAGGGAAATACTACTCACC | InDel |
|  | P5F | AGGCTTGGTTTACCGTGTTTC | InDel |
|  | P5R | GGGAGCAGACCGAAAACTAC | InDel |
|  | P6F | ATGGAATAATCATAAAGTGG | InDel |
|  | P6R | TGAGGAGGTGAAGTCCTT | InDel |
|  | P7F | GCTCTGCATGTACACGCT | InDel |
|  | P7R | GTATACTATAATGGTCTG | InDel |
|  | P8F | CGGCAACAAGAGGAGGAT | InDel |
|  | P8R | ACAGCAATGTCCGCAACGGTTTACA | InDel |
| Detecting the mutation in *acl83* | ACL83-sqF | GTGACCTGCACATACTCC |  |
|  | ACL83-sqR | TCTTGCTTCCATATGGAG |  |
| Identification of complementation lines | ACL83-1F | GTGACCTGCACATACTCC |  |
|  | AcR1 | GCGATCATAGGCGTCTCG |  |
| Detecting knockout sites of *ACL83* | ACL83KO-sqF | CTATCAACAATTCCGGCACAT |  |
|  | ACL83KO-sqR | TTCCCTTCAACAGATTACATC |  |
| Detecting knockout sites of *ROC5* | ROC5KO-sqF | AACTGGAGCGGCACGAGA |  |
|  | ROC5KO-sqR | GCACGGTAGGAAGGAGTGAAGA |  |
| Construction of complementation vector | PActin-ACL83F | CCTCTAGAGTCGACCTGCAGATGGCGGTGGGGGGAGCCGG | PstI |
|  | PActin-ACL83R | GAGCCCTGGCATGCCTGCAGTCATGCATAAGCCAGCAAAG | PstI |
| Construction of overexpression vector | 2300-35S-ACL83F | CGGGGATCCTCTAGAGTCGACATGGCGGTGGGGGGAGCCGG | SalI |
|  | 2300-35S-ACL83F | CACCATGGTACTAGTGTCGACTCATGCATAAGCCAGCAAAGTAG | SalI |
| Subcellular localization | ACL83-GFPCF | CGGGGATCCTCTAGAGTCGACATGGCGGTGGGGGGAGCCGG | SalI |
|  | ACL83-GFPCR | CACCATGGTACTAGTGTCGACTGCATAAGCCAGCAAAGTAG | SalI |
| Yeast one-hybrid | Y1H-ROC5F | GATTATGCCTCTCCCGAATTCATGAGCTTTGGGGGCCTCT | EcoRI |
|  | Y1H-ROC5R | CTTCTCGAGTCGGCCGAATTCTCAGGCGTCGCACTGCAG | EcoRI |
|  | pLaczi-ACL83pF | CGGGGATCTGTCGACCTCGAGCATACTGAGCTGTCACCATCAGCG | XhoI |
|  | pLaczi-ACL83pR | ATACAGAGCACATGCCTCGAGATCCCCGCCTGATCGAGC | XhoI |
| LUC reporter analysis | 2300-ROC5F | GGACAGGGTACCCGGGGATCCATGAGCTTTGGGGGCCTCT | BamHI |
|  | 2300-ROC5R | AGTGTCGACTCTAGAGGATCCGGCGTCGCACTGCAGCGC | BamHI |
|  | 0800-ACL83pF | GTCGACGGTATCGATAAGCTTCATACTGAGCTGTCACCATCAGCG | HindIII |
|  | 0800-ACL83pR | CGCTCTAGAACTAGTGGATCCATCCCCGCCTGATCGAGC | BamHI |
| Protein expression | pCold-TF-ROC5F | CTCGGTACCCTCGAGGGATCCATGAGCTTTGGGGGCCTCT | BamHI |
|  | pCold-TF-ROC5R | AGACTGCAGGTCGACAAGCTTTCAGGCGTCGCACTGCAG | HindIII |
| EMSA | ACL83-J-EMSA5BF | BiotinTATACTCGGAAAGTATTTCAATACCATAAATGTGTAAAGAAGAGTATTCTAAATAAAGT | Probe with Biotin |
|  | ACL83-J-EMSAF | TATACTCGGAAAGTATTTCAATACCATAAATGTGTAAAGAAGAGTATTCTAAATAAAGT | Probe without Biotin |
|  | ACL83-J-EMSAR | ACTTTATTTAGAATACTCTTCTTTACACATTTATGGTATTGAAATACTTTCCGAGTATA | Probe without Biotin |
| DAP-qPCR | ACL83-DAP-AF | GCGGCGAGAAAATGCCGCAA |  |
|  | ACL83-DAP-AR | ATCCCCGCCTGATCGAGCTC |  |
|  | ACL83-DAP-BF | CTGAAACCCACACACAAACA |  |
|  | ACL83-DAP-BR | TTGCGGCATTTTCTCGCCGC |  |
|  | ACL83-DAP-CF | GATGCTAACCCCAACCAATA |  |
|  | ACL83-DAP-CR | TGTTTGTGTGTGGGTTTCAG |  |
|  | ACL83-DAP-DF | TGCAATATCTCTTTGTTGGG |  |
|  | ACL83-DAP-DR | TATTGGTTGGGGTTAGCATC |  |
|  | ACL83-DAP-EF | CTCCAAACGGAATTGGACTG |  |
|  | ACL83-DAP-ER | CCCAACAAAGAGATATTGCA |  |
|  | ACL83-DAP-FF | ATAACGACCGTCAACAACTG |  |
|  | ACL83-DAP-FR | CAGTCCAATTCCGTTTGGAG |  |
|  | ACL83-DAP-GF | TGCATAAGTCTGATCGTCAA |  |
|  | ACL83-DAP-GR | CAGTTGTTGACGGTCGTTAT |  |
|  | ACL83-DAP-HF | TCATTGGGGAGCAATCCTGGA |  |
|  | ACL83-DAP-HR | TTGACGATCAGACTTATGCA |  |
|  | ACL83-DAP-IF | TTCCGGACTCCCGATTTCTA |  |
|  | ACL83-DAP-IR | TCCAGGATTGCTCCCCAATGA |  |
|  | ACL83-DAP-JF | GTAGACATTCATATACTCGG |  |
|  | ACL83-DAP-JR | TAGAAATCGGGAGTCCGGAA |  |
|  | ACL83-DAP-KF | GACACCACGTCTAAACTACC |  |
|  | ACL83-DAP-KR | CCGAGTATATGAATGTCTAC |  |
|  | ACL83-DAP-LF | CATACTGAGCTGTCACCATC |  |
|  | ACL83-DAP-LR | GGTAGTTTAGACGTGGTGTC |  |
|  | UBQ5-DAPF | TCGGAGACCGTGCTAGGTTT |  |
|  | UBQ5-DAPR | GCCAGCGCCCATCGATT |  |
| qRT-PCR | q-*ACL83*F | ACAGTGGAATTTGGTCGCTCA |  |
|  | q-*ACL83*R | AAGTGGACCAGCTTGCATGA |  |
|  | q-*ROC5*F | CAGGAGATGACCCAGATCGC |  |
|  | q-*ROC5*R | ACCGGAGCGTACACAACAAT |  |
|  | *Actin 1*F | TGTATGCCAGTGGTCGTACCA |  |
|  | *Actin 1*R | CCAGCAAGGTCGAGACGAA |  |
